# Determining the clinical and cost-effectiveness of nasal sprays and a physical activity and stress management intervention to reduce respiratory tract infections in primary care: A protocol for the ‘Immune Defence’ randomised controlled trial

**DOI:** 10.1371/journal.pone.0285693

**Published:** 2023-07-14

**Authors:** Jane Vennik, Adam W. A. Geraghty, Kate Martinson, Lucy Yardley, Beth Stuart, Michael Moore, Nick Francis, Alastair Hay, Theo Verheij, Katherine Bradbury, Kate Greenwell, Laura Dennison, Sian Williamson, James Denison-Day, Ben Ainsworth, James Raftery, Shihua Zhu, Christopher Butler, Samantha Richards-Hall, Paul Little

**Affiliations:** 1 Primary Care Research Centre, University of Southampton, Southampton, United Kingdom; 2 School of Psychology, University of Southampton, Southampton, United Kingdom; 3 School of Psychological Science, University of Bristol, Bristol, United Kingdom; 4 Pragmatic Clinical Trials Unit, Queen Mary University of London, London, United Kingdom; 5 Centre for Academic Primary Care, Bristol Medical School, Population Health Sciences, University of Bristol, Bristol, United Kingdom; 6 Julius Center for Health Sciences and Primary Care, University Medical Center Utrecht, Utrecht, Netherlands; 7 Nuffield Department of Primary Care Health Sciences, University of Oxford, Oxford, United Kingdom; GERMANY

## Abstract

**Background:**

Most adults in the UK experience at least one viral respiratory tract infection (RTI) per year. Individuals with comorbidities and those with recurrent RTIs are at higher risk of infections. This can lead to more severe illness, worse quality of life and more days off work. There is promising evidence that using common nasal sprays or improving immune function through increasing physical activity and managing stress, may reduce the incidence and severity of RTIs.

**Methods and design:**

Immune Defence is an open, parallel group, randomised controlled trial. Up to 15000 adults from UK general practices, with a comorbidity or risk factor for infection and/or recurrent infections (3 or more infections per year) will be randomly allocated to i) a gel-based nasal spray designed to inhibit viral respiratory infections; ii) a saline nasal spray, iii) a digital intervention promoting physical activity and stress management, or iv) usual care with brief advice for managing infections, for 12 months. Participants will complete monthly questionnaires online. The primary outcome is the total number of days of illness due to RTIs over 6 months. Key secondary outcomes include: days with symptoms moderately bad or worse; days where work/normal activities were impaired; incidence of RTI; incidence of COVID-19; health service contacts; antibiotic usage; beliefs about antibiotics; intention to consult; number of days of illness in total due to respiratory tract infections over 12 months. Economic evaluation from an NHS perspective will compare the interventions, expressed as incremental cost effectiveness ratios. A nested mixed methods process evaluation will examine uptake and engagement with the interventions and trial procedures.

**Trial status:**

Recruitment commenced in December 2020 and the last participant is expected to complete the trial in April 2024.

**Discussion:**

Common nasal sprays and digital interventions to promote physical activity and stress management are low cost, accessible interventions applicable to primary care. If effective, they have the potential to reduce the individual and societal impact of RTIs.

**Trial registration:**

Prospectively registered with ISRCTN registry (17936080) on 30/10/2020.

**Sponsor:**

This RCT is sponsored by University of Southampton. The sponsors had no role in the study design, decision to publish, or preparation of the manuscript.

## 1 Introduction

### 1.1 Background and rationale

Most adults in the UK experience at least one viral respiratory tract infection (RTI) per year [1]. Whilst symptoms are often self-limiting, they can significantly affect health-related quality of life and are the most common reason for sickness absence [2]. The majority of patients attending their GP with RTIs are prescribed antibiotics [3, 4] and primary care antibiotic use is strongly related to the threat of antibiotic resistance [5]. Individuals with comorbidities and/or recurrent infections are a higher initial priority: they have more days of illness, more severe illness, worse quality of life and higher work absence [6–8]. Low cost, non-prescription interventions to reduce severity and duration of illness associated with RTIs are needed to reduce burden on healthcare services and antibiotic usage.

#### Reducing illness through modification of the nasal environment

Antiviral nasal sprays are commonly available to purchase over-the-counter in the UK. As medical devices, they have the potential to change the nasal environment, by creating a physical barrier to viral penetration or reducing viral replication [9]. Trials of antiviral nasal sprays compared to saline suggest there may be benefit in reduction of days of symptoms when used early in the illness, but the trial evidence is rather limited [10, 11]. Saline may also have a role in reducing symptoms associated with RTIs by irrigating the nasal passages and reducing levels of virus in the nasopharynx [9]. A systematic review of saline nasal irrigation (nasal rinses and sprays) in acute upper RTIs found some benefit in symptom control, although trials were small with a high risk of bias [12]. Whilst saline nasal sprays may have some effect in managing RTIs, they make for a useful comparison for the antiviral nasal spray as they have an equivalent method of delivery.

#### Reducing illness through improving immune function

Physical activity and stress management both improve immune function and have empirical evidence of benefit in reducing illness episodes. A Cochrane review [13] of the effect of physical activity on reducing illness episodes suggests promising effects for recurrence and symptom days, although most were small trials of generally low quality. One high quality small US trial documented a reduction in illness of more than 3 days [14] and these results are supported by a more recent trial [15]. There are similar estimates from a cohort study among 1002 adults [16]. Reasons for low uptake and adherence to exercise in patients with chronic illnesses is multi-faceted [17], so simple generic advice is unlikely to work.

Perceived stress [18], negative emotion [19], and poor social support [20] predict subsequent illness, viral shedding, cytokine activity, as well as adverse mucosal defence and pathogenicity [21, 22]. Mindfulness can reduce stress and negative emotions [23]. A small US trial of an 8 week course documented a reduction of 3–4 illness days compared with controls [14] and the most recent trial [15] by the same group showed a reduction of 1 day. However, the US trials involved either intensive supervised exercise (8 sessions) or similarly intensive supervised mindfulness courses (again 8 sessions), each session being at least 2.5 hours. For behavioural and psychological support to be viable care offerings for those with RTIs in primary care, accessible and scalable delivery methods are necessary. Research has found that digital interventions which support physical activity can improve exercise outcomes for people with chronic health conditions, especially if underpinned by behavioural change theory [24]. Similarly, digital interventions supporting stress management have shown benefit in a range of health conditions [25, 26] and are implementable in the primary care setting. As yet, none have been developed and evaluated for managing RTIs.

In the present trial, we sought to determine the clinical and cost effectiveness of differing, low-cost approaches to supporting those with RTIs in primary care; nasal spray interventions and digital interventions promoting physical activity and stress, compared to usual care with brief advice for managing RTIs.

## 1.2 Objectives

### 1.2.1 Primary objective

The primary objective is to assess the effectiveness and cost-effectiveness of a gel-based nasal spray, a saline nasal spray, and a physical activity and stress management intervention, in reducing the duration of illness days due to RTIs among at-risk individuals when compared to usual care with brief advice about managing RTIs at 6 months.

### 1.2.2 Secondary objective

The secondary objectives are:

- To evaluate the incidence of all RTIs, incidence of COVID-like infections, days with symptoms moderately bad or worse; days where work/normal activities were impaired; contact with health service; hospital admissions; antibiotic usage; beliefs about antibiotics; and intention to consult, over 6 and 12 months
- To evaluate patient engagement with the interventions by exploring patients’ experiences of the different interventions to understand why different patients did or didn’t engage with the treatment/intervention, and what might affect future engagement.

### 1.3 Trial design

Immune Defence is an open, randomised, parallel group, 4-arm trial evaluating i) a gel-based nasal spray, ii) a saline nasal spray, iii) a physical activity and stress management intervention and iv) usual care plus brief advice, in reducing duration and severity of RTIs in UK primary care, over 6 and 12 months. We will assess the impact overall and in at-risk subgroups of patients defined by whether they have a) recurrent infections, no risk factors; b) risk factors, no recurrent infections; or c) risk factors plus recurrent infections.

A nested mixed methods process evaluation will triangulate qualitative interview data with quantitative attitudinal and behavioural process measures and quantitative intervention usage data.

## 2 Materials and methods

**Fig 1:**
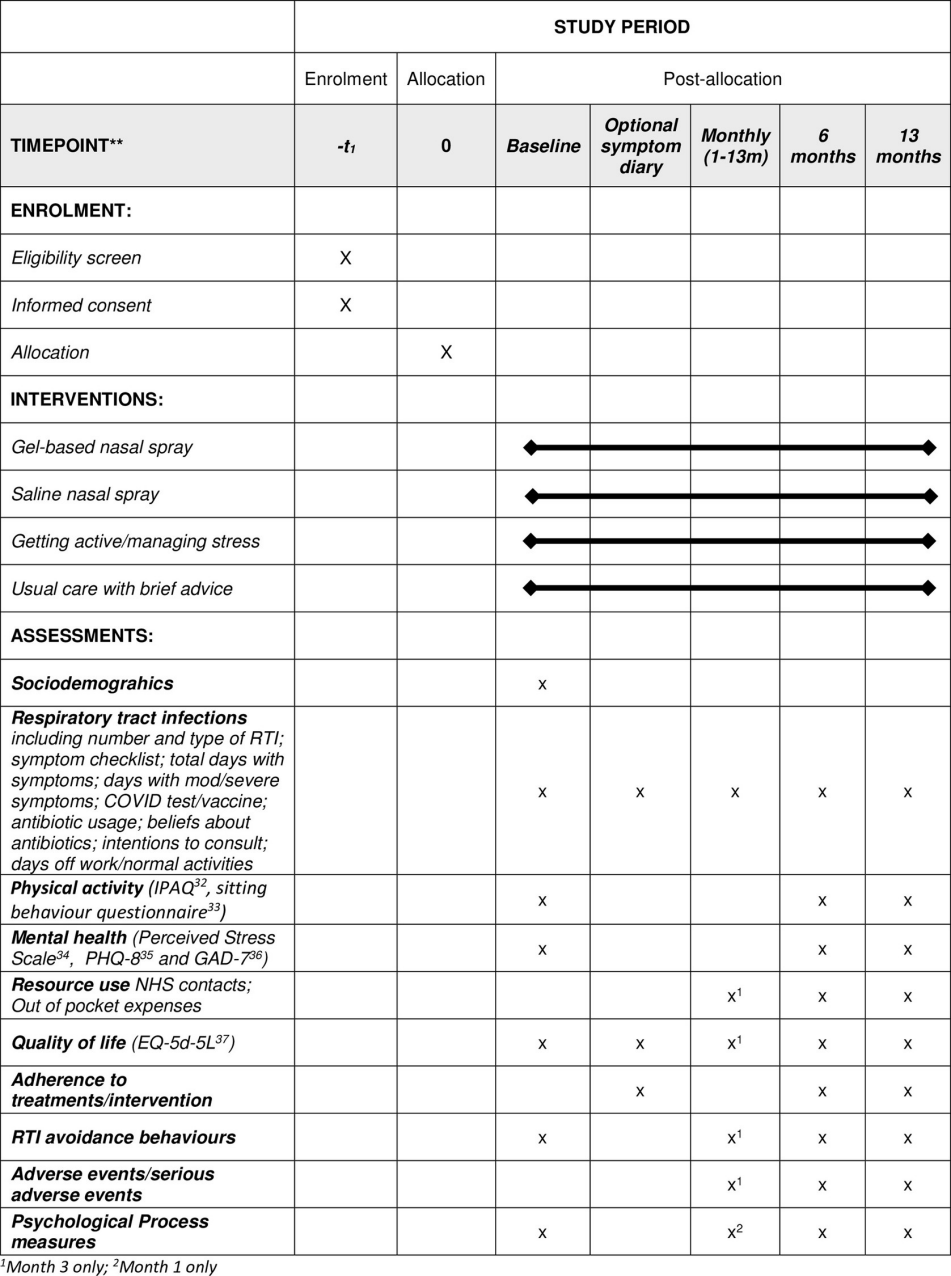
Schedule of enrolment, interventions, and assessments.

The protocol was prospectively registered with ISRCTN registry (17936080) on 30/10/2020 and is reported according to the SPIRIT guidelines (S1 Appendix). The trial schedule is reported in Fig 1.

For further information about trial registration, see Table 1.

**Table 1:** Items from the World Health organisation trial registration data set.

Data category	Information
Primary registry and trial identifying number	ISRCTN17936080
Date of registration	30/10/2020
Secondary identifying numbers	-
Source of monetary or material support	NIHR-Programme Grants for Applied Research (PGfAR) (NIHR) (UK)
Primary sponsor	University of Southampton, rgoinfo@soton.ac.uk
Secondary sponsor (s)	-
Contact for public queries	Dr Jane Vennik, j.vennik@soton.ac.uk
Contact for scientific queries	Dr Jane Vennik, j.vennik@soton.ac.uk
Public title	Evaluating nasal sprays and physical activity/stress management in reducing respiratory infections in primary care
Scientific title	Reducing respiratory infections in primary care: the Immune Defence Study
Countries of recruitment	England, United Kingdom
Health condition or problem studied	Respiratory
Interventions	1. Gel-based nasal spray (medical device) 2. Saline nasal spray (medical device) 3. Support for physical activity and stress management (behavioural intervention) 4. Usual care for infections
Key inclusion and exclusion criteria	Inclusion criteria: 18+ years with a serious comorbidity or a risk factor for infection: Immunosuppression due to a serious illness or medication (e.g. chemotherapy); heart disease; asthma or lung disease; diabetes; mild hepatic impairment; stroke or other severe neurological condition; obesity (BMI>30); aged >65; 3 or more RTIs in the last year Exclusion criteria: a terminal illness/palliative care; dementia; residential care; pituitary adenoma; pregnant/breast-feeding; regularly use nasal sprays to prevent respiratory infections; are allergic to nasal sprays; live in the same household as another participant; involved in the development phase of the trial.
Study type	Randomised controlled trial
Date of first enrolment	12/12/2020
Target sample size	Up to 15000
Recruitment status	Recruiting
Primary outcomes	Total number of days of illness due to respiratory tract infections over 6 months.
Key secondary outcomes	Days with symptoms moderately bad or worse; days where work/normal activities were impaired; incidence of RTI; incidence of COVID-19; health service contacts; antibiotic usage; beliefs about antibiotics; intention to consult; number of days of illness in total due to respiratory tract infections over 12 months

### 2.1 Trial setting

Participants will be identified through UK primary care. GP practices will be engaged through the NIHR Clinical Research Networks, and selected from regions of urban and rural settings, large and small practices, and high and low deprivation.

### 2.2 Eligibility criteria

Participants will be eligible for the trial if they are 18 years and above, with a serious comorbidity or a risk factor for infection and normally experience one or more RTIs per year: Immunosuppression due to a serious illness or medication (e.g. chemotherapy); heart disease; asthma or lung disease; diabetes; mild hepatic impairment; stroke or other severe neurological condition; obesity (BMI>30); aged >65; 3 or more RTIs in the last year.

Participants will be excluded if they have any of the following identified through practice records: a terminal illness or are receiving palliative care; have a diagnosis of dementia; live in residential care; have pituitary adenoma. Participants will also be excluded through participant self-report if they: are pregnant/breast-feeding; regularly use nasal sprays to prevent respiratory infections; are allergic to nasal sprays; live in the same household as another participant; have been previously involved in the development phase of the trial.

### 2.3 Interventions

#### 2.3.1 Active treatment arms

Participants in the three active treatment arms will have access to a digital intervention (website available via desktop and smart devices) which has been developed using evidence-, theory- and person-based approaches [27]. Each intervention aims to facilitate behaviour change through three key mechanisms: (1) promotion of positive outcome expectancies (beliefs that the behaviour will prevent RTIs); (2) improved self-efficacy for performing the behaviour (mainly through clear instructions on how to perform the behaviour and advice on overcoming behavioural barriers); and (3) reduction of behavioural concerns (e.g. safety). The interventions include persuasive information about how each individual intervention may help reduce the number, duration and severity of RTIs experienced, clear instructions about how to perform the target intervention behaviour, and information to address key concerns that participants may have about engaging in the behaviour. Intervention-specific behaviour change techniques are discussed below.

**Nasal sprays**

The two nasal sprays in the trial are available to purchase over-the-counter in pharmacies and supermarkets in the UK and are registered as medical devices: a gel-based nasal spray and a saline nasal spray. Full details of the nasal spray constituents will be presented when the findings are published, to preserve masking during the trial period.

The digital interventions supporting use of the nasal sprays will provide explanations and instructions on how to use the spray, including a demonstration video. Concerns about using the sprays will be addressed, and tips to help people to remember to use and to adopt the correct technique are included. This information is duplicated in a paper booklet that will be sent to participants together with 2 bottles of nasal spray after randomisation (further bottles will be available on request). The digital intervention to support nasal spray use and its development is described in more detail elsewhere [28].

Participants will be instructed to use the nasal spray in three ways during the trial:

- **At first signs of an infection:** Up to 6 times daily (2 sprays in each nostril) until symptom-free for 2 days.
- **After potential exposure to infection** (e.g. using public transport, supermarkets, cafes/pubs): 2 sprays in each nostril immediately after exposures, 1 hour later and last thing at night.
- **After prolonged exposure** (e.g. close contact with/living with someone who has an infection): Up to 6 times daily (2 sprays in each nostril) until the close contact has recovered.

**Digital intervention promoting physical activity and stress management**

The digital intervention promoting physical activity and stress management includes a brief introductory section with content on the impact of RTIs and how physical activity and stress management can prevent RTIs, followed by two previously developed online modules to support physical activity *(Getting Active)* and stress management *(Healthy Paths through Stress)*.

The ‘Getting Active’ was developed as part of a digital intervention to improve overall quality of life among cancer survivors [29, 30] and has now been optimised for people at risk of RTIs. ‘Getting active’ aims to build motivation for physical activity by promoting the benefits and addressing concerns, increasing self-efficacy by providing advice on safe physical activity and overcoming barriers, and facilitating behavioural regulation through goal setting, goal review (automated tailored feedback and support), action planning and self-monitoring (using pedometers to count and record steps). Getting Active sends automated emails encouraging and supporting participants to engage with the intervention and overcome barriers to physical activity. As well as the addition of the introductory section, the intervention was adapted for people at risk of RTIs by including physical activity benefits and addressing concerns relevant to this target group. A pedometer will be sent out by post to each participant at the recruitment stage.

“Healthy Paths through Stress” was initially developed as a digital intervention for stress/emotional distress for patients in primary care [31]. It has also been used to manage distress in cancer survivors [29]. Participants can explore a range of evidence-based techniques and read rationales and instructions for trying them. The techniques in the intervention are drawn from behavioural activation (pleasant activity scheduling, sleep hygiene) and/or mindfulness-based approaches (e.g. 3-minute breathing space, self-compassion exercise). Participants can select those they find most helpful. Participants are advised that the techniques can be helpful at any time but are particularly useful when experiencing challenging life events.

Participants are encouraged to try both interventions and will have access to them at all times throughout the trial. These are complex interventions which could plausibly generate additive effects of the individual components.

#### 2.3.2 Comparator: Usual care with brief advice about managing infections

Participants allocated to usual care will receive a brief page of advice about managing respiratory tract infections, based upon NHS current advice (rest, keeping warm, fluids, over-the-counter medications for symptom relief). Specific resource use will be tracked for health economic analysis. The usual care group will be asked not to use any over-the-counter nasal sprays during the trial period. After 12 months, participants will be offered access to the digital intervention promoting physical activity and stress management for a short time.

### 2.4 Outcome measures

#### 2.4.1 Primary outcome

The primary outcome will be the total number of days of illness due to respiratory tract infections over 6 months.

#### 2.4.2 Key secondary outcomes

Key secondary outcomes include: days with symptoms moderately bad or worse; days where work/normal activities were impaired; incidence of RTI; incidence of COVID-19; health service contacts; antibiotic usage; beliefs about antibiotics; intention to consult over 6 and 12 months; number of days of illness in total due to respiratory tract infections over 12 months

#### 2.4.3 Other secondary outcomes

Other secondary outcome measures will be as follows:

- Physical activity will be evaluated using International Physical Activity questionnaire [32] sitting behaviours questionnaire [33] and activity monitors.
- Mental health will be assessed using the Perceived Stress Scale [34], PHQ-8 [35] and GAD-7 [36]
- Participant quality of life will be determined using EQ-5d-5L [37]
- Resource use: NHS contacts will be collected through participant self-report and by retrospective notes review. Out-of-pocket spending will be collected through participant self-report RTI related medication collected through retrospective notes review.
- Side effects of the nasal sprays will be collected through participant self-report
- Serious adverse events will be collected through participant self-report and reports from the GP surgery.
- Adherence to nasal sprays, getting active and healthy paths assessed through participant self-report.
- RTI avoidance behaviours/Vitamin D supplementation assessed through participant self-report.

### 2.5 Participants

**Fig 2:**
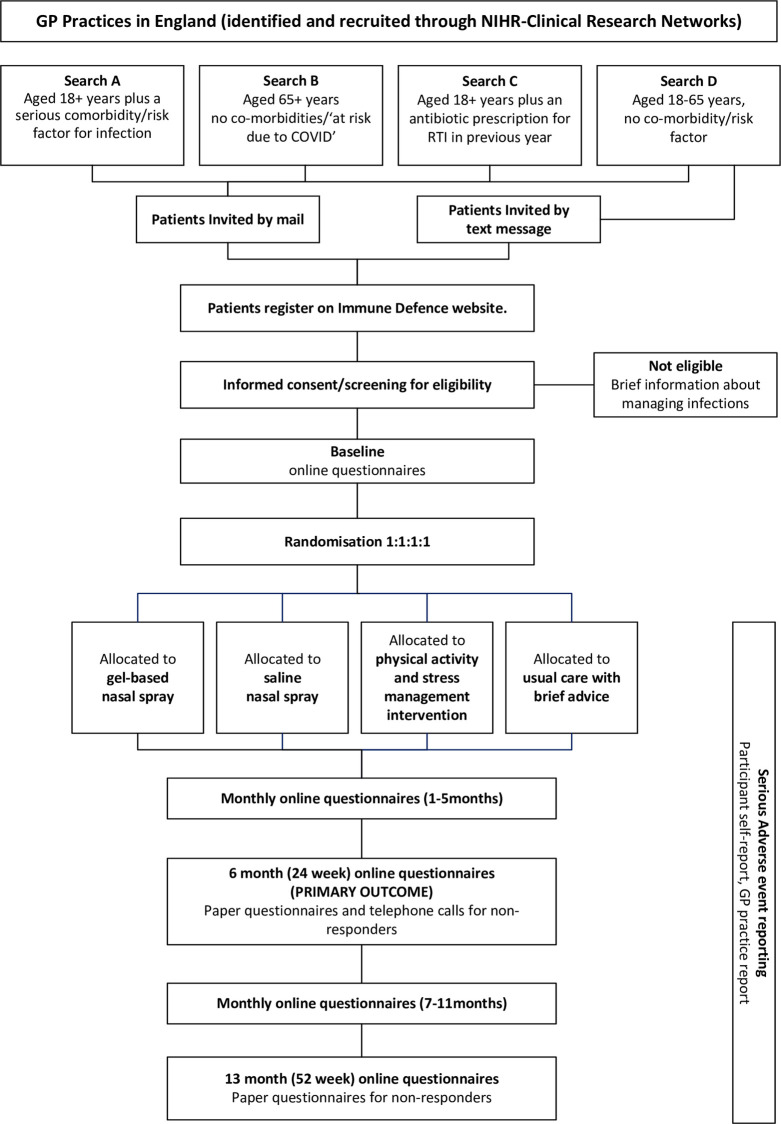
Participant flow chart.

Participant flow chart is presented in Fig 2. Recruitment commenced in December 2020 and is expected to complete by April 2023.

#### 2.5.1 Participant identification

Participants will be invited through targeted invitation by the GP practice. Practices will use executable files to identify eligible participants from the clinical record system. Lists of patients will be generated as follows:

Search A: Aged 18+ years plus a serious comorbidity/risk factor for infection *(all seasons)*
Search B: Aged 65+ years, no comorbidities/‘at risk due to COVID’ *(season 1 only)*
Search C: Aged 18+ years plus an antibiotic prescription for RTI in the previous year to identify those most likely to be at risk of recurrent RTIs *(all seasons)*
Search D: Aged 18–65 years, no comorbidities/risk factors–a wide sample to identify younger participants who may self-report recurrent infections *(season 3 only)*

Lists will be checked by the practice to ensure suitability to receive an invitation. A random sample of participants (up to 3000 letters or 6000 texts per practice depending on practice list size) will be sent an invitation pack by either i) a secure mail service (Docmail) containing an invitation letter, participant information sheet, details about how to sign up, and a sign-up code to facilitate stratification by health status, or ii) a brief text message invitation with links to receive an invitation pack by email.

#### 2.5.2 Informed consent and screening

Interested participants will visit the Immune Defence website and sign up using their unique sign-up code. Participants will give their consent online prior to completing the screening questions to check for exclusion criteria not identified by the practice searches (*pregnant/breast-feeding; regularly use nasal sprays to prevent infections; allergy to nasal sprays; living in the same household as another participant; previously involved in the development phase of the trial*). Ineligible patients will be provided with a link to a brief advice page about managing RTIs. Patients who meet the screening criteria will complete baseline measures and proceed to randomisation.

#### 2.5.3 Randomisation

The randomisation process (1:1:1:1) for this trial will be fully automated. The Immune Defence website software will generate a randomisation sequence and computer algorithm to block randomise participants to the 4 trial groups. As randomisation is automated, the sequence will be concealed from the trial team. Patients will be stratified on the basis of whether they are in a higher risk group (over 65 and/or having comorbid condition) and whether or not they have recurrent RTIs (≥3 in the last year) to three strata: stratum 1 (recurrence, no risk factors); stratum 2 (risk factors, no recurrence); stratum 3 (risk factors plus recurrence).

#### 2.5.4 Blinding/masking

Full blinding of trial participants to their intervention group is not possible. However, to reduce possible contamination (as the nasal sprays are available to purchase over-the-counter in pharmacies and supermarkets) the nasal sprays will be masked to their content by removing the manufacturers labels, and adding generic trial labels. As the nasal sprays in the study are medical devices there should not be a need to unmask them during the study. The GP practices are aware of the contents and other medical professionals involved in the care of participants can contact the study team for the same information if required. Staff responsible for data entry and data analysis will be blind to the treatment group.

### 2.6 Sample size

Our primary outcome is the number of days of illness in total due to RTIs at 6 months.

**Provisional sample size calculation agreed with the funder following the start of the COVID pandemic:**

To detect a 1-day difference among individuals having an infection (hazard ratio 1.2) for alpha of 0.01 and 90% power, we estimated we would require 147 individuals per group, and allowing for at least 15% of individuals to contract an infection during a 6-month winter/spring period 980 individuals per group. With four groups and 80% follow-up, then 4900 individuals would be needed. We also aim to estimate outcomes in the three strata (stratum 1 –recurrence, no risk factors; stratum 2—risk factors, no recurrence; stratum 3—risk factors plus recurrence) so planned to recruit 14,700 participants in total. We accepted that this would provide less power for our key secondary outcome (incidence of infections). However, the aim for the latter outcome is to compare each intervention with control, so using the arguments of Cook and Farewell, since each analysis of intervention versus control is independent, this should not require a conservative Bonferroni correction [28], and we can use an alpha of 0.05. Using these assumptions, the above sample size would provide more than 80% power to estimate a 25% reduction in the incidence of infections from 20% to 15% in each stratum and more than 90% power if the incidence of infections is 15% using all strata combined. We will update the sample size calculations based on the observed rates of infections in each season when the SAP is drafted.

**Revised estimates for the primary outcome based on data of the incidence of infections from the first two seasons (2020/21; 2021/22):**

1. Stratum 1 (recurrence, no risk factors): Currently, in this group about 71%, compared to our original assumption of 15%, had an infection. Basing our recalculation on the more conservative lower limit of the 95% confidence interval of this estimate, we might assume that at least 65% of this group get an infection. Based on the original number per group of 147 (i.e. changing no other assumptions), we’d need 226 per group, and for 4 groups and with 80% follow up 1130. For 80% power, based on the original assumptions, we would require 111 per group. If 65% get an infection, then we would need 111/0.65 = 171 per group. For 4 groups with 80% follow up we would require 855 participants.
2. Stratum 2 (risk factors, no recurrence): In this group, the observed infection rate has been around 40% compared to our assumption of 15%. On this basis, based on the original sample size calculation of 147 per group, we would require 147/0.40 = 368 per group. With 4 groups and 80% follow up this gives a total of 1472 for 90% power. For 80% power, we would require 1388 participants.
3. Stratum 3 (risk factors plus recurrence): The infection rate is currently around 62% and follow up is just under 80%. On the basis of the original calculation, assuming at least 60% having an infection, we would need 245 per group, time 4 groups with 80% follow up gives 1225.

**Revised estimates for the number of infections (secondary outcome):**

1. In stratum 1 (recurrence, no risk factors): Assuming an infection rate of 65% and for 15% absolute reduction to 50% we require 227 complete cases in each group for 90% power (1135 with four groups and 80% follow up) and 170 for 80% power (850 in total)
2. In stratum 2 (risk factors, no recurrence): To detect a change from 40% to 30% we estimate we need 476 compete cases in each group for 90% power (2380 with four group and 80% follow-up) and 356 for 80% power (1780 in total).
3. In stratum 3 (risk factors plus recurrence): To detect a change from 60% to 45% for 90% power we need 231 complete cases in each group or 1155 for four groups and 80% follow-up.

## 2.7 Data collection and management

### 2.7.1 Data collection

Data will be collected online at baseline, and then monthly for 13 months (52 weeks) using the trial and intervention website. Each month participants will receive an automated email and 1 week reminder to complete brief questionnaires, with more detailed questions at 6 months and 13 months. Non-completers at 6 months and 13 months will also be sent a paper questionnaire after 2 weeks and at 6 months (primary outcome) will also be followed up by telephone for limited responses after 3–4 weeks. Willing participants will complete an optional daily paper-based symptom diary should they develop an RTI during the trial period.

All data will be managed in accordance with the General Data Protection Regulation (GDPR) 2018. Electronic data will be stored on secure servers at University of Southampton and password protected. Anonymised paper questionnaires and diaries will be stored in locked filing cabinets prior to data entry and destroyed at the end of the study. Personal data will be collected for the purposes of study participation and kept separately from the questionnaire data for 10 years after the study has completed. Anonymous study questionnaires will be deposited in a secure data archive and made available for secondary data analysis, subject to further ethical approvals.

### 2.7.2 Data analysis

A detailed statistical analysis plan will be developed prior to data analysis. IBM® SPSS® software platform, Stata and Excel software will be used to evaluate outcomes.

The primary analysis will be based on those who report at least one infection in a normal year (before COVID). We will also conduct a sensitivity analysis to include all participants who included under the broader inclusion criteria (‘at-risk’ due to COVID).

The primary time point for analysis will be at 6 months. The analysis of the primary outcome and other continuous outcomes will compare all groups. For the incidence of infection data the primary analysis will be between each intervention group and usual care, and if any of these demonstrate an intervention is effective then we propose secondary comparisons between groups. A secondary analysis will use repeated measures over the year.

The particular regression models used will depend on the data and the patterns of residuals but we anticipate logistic regression models for dichotomous outcomes, negative binomial models for count data, and generalised linear mixed models will be used for continuous variables (all controlling for baseline values; stratification variables and potential confounding variables). Intention to Treat (ITT) analysis with missing data imputed (via chained-equations multiple imputation model) will be the primary analysis, and complete cases as a sensitivity analysis. Secondary analyses will follow a similar modelling approach to the primary analyses. The repeated measures analysis over the one-year period will allow for the clustering of observations within participants over time. Estimates will be provided for key subgroups (e.g. those with recurrent infections (>3/year), age >65, the presence and number of serious comorbidities). Results will be reported in line with the CONSORT guidelines.

## 2.8 Economic evaluation

The economic analysis will take a NHS perspective covering the intervention costs, NHS and personal social service (PSS). The impact on cost effectiveness of out-of-pocket spending and employment will be assessed. Data will be collected by patient monthly report, supplemented by a brief case notes review for non-responders, and a detailed notes review on 5–10% of participants to check precision of self-reported data. Quality of Life (QOL) will be measured by EQ-5D-5L at baseline, 3 months 6 months and 12 months. QALYs will be estimated by means of area under the curve. The differences for Cost and QALYs between interventions will be estimated adjusted for baseline characteristics. Where appropriate we will estimate incremental cost-effectiveness ratios (ICERs) for comparing different interventions. Bootstrapping will generate incremental cost effectiveness ratios (ICERs). Cost-effectiveness acceptability curves will be produced to reflect the probability of an intervention being cost-effective at different willingness-to-pay values per QALY gained. Major assumptions made in the analysis will be tested by means of sensitivity analyses.

## 2.9 Process evaluation

We will conduct a mixed-methods process evaluation to examine uptake and engagement with the trial interventions and trial procedures and explore potential mechanisms of action and contextual factors that may influence intervention delivery and outcomes.

**Qualitative:** A purposive sample of up to 100 patient participants (final numbers to be determined considering information power [38]) will be invited to take part in a semi-structured telephone interview at least 3 months after randomisation each year/phase of the trial. The sample will include participants from each of the four intervention arms; and a range of ages, genders, ethnicities, deprivation level, education, risk factors for infections, history of RTIs, self-reported beliefs about RTIs, self-reported behaviour (physical activity, stress management, nasal spray use), and intervention usage. An interview topic guide was developed to explore participants’ experiences of the intervention and the trial processes and procedures, identify barriers and enablers to intervention engagement, perceived benefits of the intervention, impact of the COVID-19 pandemic on their preventative behaviours and intervention use. Interviews will be transcribed verbatim and transcripts will be managed using NVivo qualitative data management software and analysed using reflexive thematic analysis [39]. We will examine how and why our qualitative findings converge with, complement, or contradict the quantitative findings.

**Quantitative:** Analysis of the process questionnaire data and usage data will assess intervention reach (uptake; sample characteristics), self-reported adherence to the nasal sprays and getting active and healthy paths interventions, predictors of adherence and outcomes (age; gender; education; comorbidities; RTI and behavioural beliefs; barriers to behaviour change. We will examine the moderator effects of baseline characteristics (particularly demographics) on intervention engagement and outcomes, and the factors likely to mediate engagement (e.g. behaviour, beliefs). We will also employ multi-level modelling to investigate how process measures relate to outcomes and intervention engagement.

## 2.10 Patient and public involvement

Two public contributors with experience and understanding of respiratory issues contributed to the overall design of the study and are full collaborators on the programme grant.

Three public contributors assisted during the development of the digital interventions used in this trial. This included taking part in think-aloud interviews, working through sections of the interventions with a researcher. This helped to refine the interview topic guides and ensure the language and communication was more broadly appropriate. Two contributors also reviewed and commented on early drafts of the intervention pages and automated emails, and reviewed the nasal spray instruction videos and written materials.

During development of the trial processes and materials, the public contributors piloted the baseline and outcome questionnaires to ensure feasibility and clarity, and have reviewed all participant-facing documentation.

Three public contributors are full members of the Trial Stakeholder group. They will attend monthly stakeholder meetings and contribute to management decisions regarding development and operationalising the trial. They will also contribute to the writing up of trial outputs including publications and lay summaries.

## 2.11 Monitoring

### 2.11.1 Data monitoring and oversight

An independent trial steering committee (TSC) will be convened to provide trial oversight. The TSC will also provide the function of the Data Monitoring and Ethics Committee (DMEC) as the interventions and the trial processes are considered low risk.

Monthly management meetings with key stakeholders including co-investigators, collaborators, trial management and public contributors will be held throughout the trial period.

### 2.11.2 Adverse event reporting

Any adverse events reported by participants or by the GP surgery will be documented and reviewed for severity, expectedness and relatedness to the trial and trial interventions. Serious adverse events (SAEs) will be reviewed for expectedness. The trial includes elderly and high-risk patients so a high rate of planned or emergency hospitalisations, new illness diagnoses, worsening of pre-existing conditions, cardiovascular events, falls, and deaths are expected in this population. These events will be documented and reviewed by the chief investigator. All SAEs including serious adverse device effects (SADEs) that are unexpected or considered *possibly*, *probably* or *definitely* related to the trial or interventions, will be reported to the sponsor and the REC immediately and followed up with a detailed written report.

### 2.11.3 Discontinuation and withdrawal of participants

If a participant expresses a wish to withdraw from the trial treatment (e.g. does not wish to use the nasal sprays or advice from the Getting Active/Healthy Paths website), the trial team will explain the importance of follow-up and ask if they would be willing to continue in the trial without using the interventions and complete the questionnaires. This will be recorded in the participants records. If the participant does not confirm continuation or requests full withdrawal, the participant will be withdrawn from the trial and receive no further communication from the trial team. All data collected up to the point of withdrawal will be used, unless the participant has specifically requested for it to be deleted.

If a participant moves to a new GP practice, the trial team will endeavour to contact the new practice and ask if they would notify the team of any SAEs during the trial period.

## 2.12 Ethics and dissemination

### 2.12.1 Research ethics approval

This trial was approved by the South East Scotland Research Ethics Committee 01 (20/SS/0102) on 23^rd^ October 2020 and the HRA on 29^th^ October 2020.

### 2.12.2 Consent

All participants will receive information and will have the opportunity to ask questions prior to deciding whether to take part. For the main trial, participants will give consent online after registering on the Immune Defence website. For participants taking part in a telephone interview, written consent or verbal consent over the telephone will be given by the participant to the researcher.

### 2.12.3 Dissemination

The results of the trial will be disseminated in peer-reviewed journals and presented at primary care conferences. All participants (GP practices and patient participants) will be sent an accessible copy of the findings by email within 6 months of trial completion (unless they have opted out of further contact). We will also disseminate our findings to the wider public via our patient collaborators, PPI panels and through social media.

Findings will report anonymised data and summary statistics. No information identifying individual participants or GP practices will be reported. Trial records will be retained for a minimum of 10 years after the trial has been published.

## 3 Discussion

RTIs generate symptoms which are the major driver of winter consultation pressures. Most are viral in aetiology, so limiting viral replication and load through use of nasal sprays, could reduce symptom burden and healthcare consultations, providing an evidence-based alternative to antibiotics. Similarly, if an intervention to promote physical activity and stress management can be shown to reduce the number and severity of infections this will provide significant motivator for those who suffer with recurrent infections to use the intervention. Providing patients with alternative strategies to antibiotics is likely to modify patient beliefs and expectation, reduce antibiotic use, and alter subsequent help seeking behaviour [6, 7, 32, 33]. This is also likely to be relevant during pandemic winters. SARS-CoV-2 is transmitted in similar ways to other respiratory viruses, thus nasal sprays that reduce viral load have the potential to reduce COVID-19 as well as usual winter illnesses. The brief physical activity and stress management intervention is likely to be as pertinent during the pandemic winters, particularly as levels of anxiety and stress will be higher, and maintaining a good level of physical activity has been strongly encouraged.

The trial has been designed to assess the impact in groups of individuals who are at high risk for infections/adverse outcomes. These include individuals who report frequent recurrent infections but with no significant comorbidities; individuals with serious comorbidities/risk factors alone; and those with both recurrent infections and comorbidities/risk factors. Thus, it is plausible that either an intervention to use a web-based applications to modify stress and increase physical activity, or alternatively advice to use an inexpensive and easily available nasal spray, could both help symptom control, de-medicalise infections, and be widely implemented among the broad group of patients suffering recurrent infections.

### 3.1 Status and timeline

This trial is funded as part of the NIHR PGfAR RECUR Programme. The development and optimisation of the trial platform was nearing completion in March 2020 and we had planned a large feasibility trial in the 2020–21 winter season followed by a full trial in the following 2021–2023 seasons. However, with our experiences of conducting similar large trials together with support from the funder, we felt able do a very much larger full trial starting in the 2020–2021 winter season. This allows us to provide information not only about whether such interventions are likely to work in a pandemic season but also in more ‘normal’ winters for respiratory illnesses.

Recruitment of participants commenced in December 2020 and is expected to be completed by April 2023. The last participant will complete the trial by end April 2024. The current version of the protocol is v8.1 dated 15-11-2022.

There have been two main amendments to the trial in response to the changing landscape of the COVID pandemic. In season 1 we included participants aged 65+ considered higher risk of COVID-19. From Season 2 onwards we tightened the inclusion criteria to include 65+ who also reported at least 1 RTI in a normal year (before COVID). We considered this would give a better chance of participants engaging with our interventions. In season 3 we are focussing recruitment on participants with comorbidity/risk factors, and not recruiting any further participants aged 65+ without any other risk factors for infection.

## Supporting information

S1 Appendix SPIRIT checklist. (DOC)

S1 File (PDF)
