# Supplementary material for: Determining the clinical and cost-effectiveness of nasal sprays and a physical activity and stress management intervention to reduce respiratory tract infections in primary care: A protocol for the ‘Immune Defence’ randomised controlled trial
Source: PLoS One. 2023 Jul 14;18(7):e0285693. doi: 10.1371/journal.pone.0285693 (PMC10348543; doi:10.1371/journal.pone.0285693)
Supplement: S1 File — (PDF) [file pone.0285693.s002.pdf]

# Protocol v8.1

(redacted)

# Reducing respiratory infections in primary care:
# The Immune Defence Study

| | |
|---|---|
| Sponsor | University of Southampton |
| Funder(s) | NIHR PGfAR: RP-PG-0218-20005 |
| ERGO number | 56474 |
| IRAS number | 288431 |
| ISRCTN | 17936080 |



| | |
|---|---|
| Professor Paul Little | Chief investigator |
| Dr Adam Geraghty | Co-chief investigator |
| **Co-applicants of the Programme Grant for Applied Research** | |
| Prof Lucy Yardley | University of Southampton / University of Bristol |
| Prof Alastair Hay | University of Bristol |
| Prof Christopher Butler | University of Oxford |
| Prof Michael Moore | University of Southampton |
| Prof Nick Francis | University of Southampton |
| Prof Theo Verheij | University of Southampton |
| Mrs Samantha Richards-Hall | University of Southampton |
| Dr Shihua Zhu | University of Southampton |
| Dr Beth Stuart | University of Southampton |
| Ms Sonia Newman | University of Southampton |
| Prof James Raftery | University of Southampton |
| Dr Ben Ainsworth | University of Bath |
| Dr Kat Bradbury | University of Southampton |
| Dr Kate Greenwell | University of Southampton |
| Dr Laura Dennison | University of Southampton |
| Dr James Denison-Day | University of Southampton |
| Dr Sian Williamson | University of Southampton |
| Ms Kate Rumsby | University of Southampton |
| Dr Jane Vennik | University of Southampton |
| Mrs Samantha Williams | University of Southampton |
| Ms Sophie Johnson | University of Southampton |
| Mrs Jane Barnett | University of Southampton |

## Contents

Contents ........ 2
Study Summary ........ 4
Lay summary ........ 6
Study flow chart ........ 7
1 Background ........ 8
1.1 Introduction: 'Non-pandemic' winters ........ 8
1.2 COVID-19 pandemic ........ 8
1.3 Simple non-specific measures to modify the physical environment of respiratory viruses: the role of temperature and pH in lowering the size of the viral inoculum ........ 8
1.4 Empirical evidence of reducing incidence and symptoms of RTIs in humans through anti-viral nose sprays in non-pandemic years. ........ 9
1.5 Reducing illness episodes through physical activity and stress management ........ 9
1.6 Progress of existing grant ........ 10
1.7 Summary ........ 10
2 Aims and objectives ........ 11
2.1 Aim. ........ 11
2.2 Objectives: ........ 11
2.2.1 Primary objective ........ 11
2.2.2 Secondary objectives ........ 11
3 Methods ........ 11
3.1 Design ........ 11
3.2 Participants ........ 11
3.2.1 Inclusion criteria ........ 12
3.2.2 Exclusion criteria ........ 13
3.3 Sample size ........ 13
4 Interventions ........ 14
i. [redacted] nasal spray ([redacted]) ........ 14
ii. Saline nasal spray ........ 15
iii. Lifestyle intervention. ........ 15
iv. Usual care with brief advice: ........ 16
5 Recruitment ........ 16
5.1 Practice recruitment ........ 16
5.2 Patient recruitment ........ 17
6 Trial procedures ........ 19
6.1 Informed consent and screening ........ 19

6.2 Baseline measures 19
6.3 Randomisation procedures 19
6.4 Blinding 20
6.5 Outcome data collection 21
6.5.1 Outcome measures 21
6.5.2 Primary outcome measure 22
6.5.3 Secondary outcome measures 22
6.5.4 Methods of data collection 23
6.6 Nested qualitative interviews 24
6.7 Recording and reporting adverse events 25
6.7.1 Definition of non-serious adverse event (AE) 25
6.7.2 Definition of serious adverse event 25
6.8 Discontinuation/study withdrawal 27
6.9 Definition of End of Study 27
7 Analysis 27
7.1 Statistical analysis 27
7.2 Qualitative process analysis 28
7.3 Quantitative process analysis 28
7.4 Health economic analysis 28
8 Dissemination plans 29
9 Patient and public involvement 29
10 Ethical issues 30
10.1 Informed consent 30
10.2 Assessment and management of risk 30
10.3 Research Ethics Committee (REC) and other Regulatory review & reports 30
10.4 Indemnity 30
11 Data Management, Data Protection, Data Security 30
11.1 Personal Data 30
11.2 Research data 31
11.3 Audio-recordings of participant interviews 31
12 References 31

## Study Summary

| | | |
|---|---|---|
| Study Title | Reducing respiratory infections in primary care: the Immune Defence Study | |
| Internal ref. no. (or short title) | Immune Defence Study | |
| Study Design | Randomised 4-arm trial | |
| | **Objective** | **How measured** |
| Primary research question | Do each of the trial interventions [redacted] nasal spray, saline nasal spray, or support physical activity and stress management) reduce the number of days in total due to respiratory tract infections (RTIs) over 6 months, when compared with usual care | Online proformas: (Self-reported RTIs using validated proformas monthly, at 3, 6 and 12 months) |
| Secondary research questions | Do each of the trial interventions reduce the number of days of illness rated moderately bad or very bad due to respiratory tract infections (RTIs) over 6 months, when compared with usual care<br><br>Do the trial interventions reduce the incidence of RTIs?<br><br>Do the trial interventions reduce health service contacts for RTIs?<br><br>Do the trial interventions reduce RTI-related hospital admissions?<br><br>Do the trial interventions reduce health service resource use (and, if so, are they cost-effective)?<br><br>Do trial interventions reduce antibiotic use for RTIs?<br><br>Do the trial interventions reduce the incidence of COVID-like illness?<br><br>Do the trial interventions reduce the incidence of confirmed COVID-19 infections? | Online proformas: (Self-reported RTIs using validated proformas monthly, at 3, 6 and 12 months)<br><br>Online proforma<br><br>Online proformas and medical record review<br><br>Online proformas and medical record review<br><br>Online proformas and medical record review<br><br>Online proformas and medical record review<br><br>Online proformas<br><br>Online proformas (reporting testing as part of normal management COVID-+ve or COVID--ve); |

| | |
|---|---|
| Inclusion criteria | • Age ≥18 years with either a comorbid risk condition or a history of 3 or more RTIs in the past year AND 1 or more RTIs in a normal year<br>• Age ≥65 years AND 1 or more RTIs in a normal year *(criterion removed for season 3*)<br>• Has access to the internet |
| Exclusion criteria | • Terminal illness/palliative care;<br>• Living with dementia<br>• Living in residential care<br>• Pregnancy or breast-feeding;<br>• Pituitary adenoma/resection.<br>• Regular use of [redacted] or similar nasal sprays for respiratory infection control in the last 6 months<br>• allergy to nasal sprays;<br>• Living in the same household as another participant<br>• Previously involved in RECUR development work |
| Intervention groups | **Patients randomised to 1 of 4 treatment groups:**<br>Microgel nasal spray [redacted]<br>Saline nasal spray<br>Lifestyle intervention (support for physical activity and stress management)<br>Usual care (brief advice) |
| Planned Size of Sample (if applicable) | 15000 |
| Follow up duration (if applicable) | 12 months |
| Planned Study Period | August 2020 – October 2023 |
| Total number of sites | Up to 200 GP practices |

## Lay summary

A range of viruses circulate each winter and cause respiratory infections (RTIs) (the viruses that causes colds, sore throats, sinus, chest or ear infections, flu). These can lead to people being off work, to seeking help from the NHS, and to be admitted to hospital in winter months. The combined effect of both the normal winter viruses (and also the COVID virus in the current pandemic) are likely to cause major problem for the NHS not only during the coming 2020-21 winter season but in subsequent years. There is promising evidence that using nasal sprays, or alternatively reducing stress and increasing exercise, could help people's immune defences, reduce the number of people getting infections, reduce how severe illnesses are and how long they last.

The NIHR has funded the RECUR Programme to develop and trial interventions to find out if they reduce the incidence of infections. We have developed a website called Immune Defence which will help us to see if using nasal sprays or getting more physically active and reducing stress can help people get fewer and less severe infections. We have involved both a range of patients and also patient representatives as collaborators in the design of our study to help ensure the procedures are relevant and appropriate to patients. We had planned a large feasibility study this year in the 2020-21 winter season, and a full trial in a 'normal' year in the 2021-2023 winter seasons. However, the stage of development this year and our previous experience of running large similar trials suggests that instead of the feasibility study we can move to a larger full trial straight away among at risk groups so that we generate a sizeable sample during the pandemic.

This study will involve approximately 200 GP practices and up to 15000 patients who are at risk from respiratory infections. Patients will be invited to take part in the study through invitation letters from their GP surgery. Those who are interested in taking part will be asked to register online and to answer some questions to ensure the study is right for them. Eligible patients will be randomised to one of the following groups for 12 months: i) A microgel nasal spray [redacted] ii) Saline nasal spray, iii) Getting Active and Reducing Stress or iv) Usual Care. Participants will be asked to complete monthly questionnaires for 12 months, and more detailed questionnaires at 3, 6 and 12 months about any infections and about their general health. Patients happy to do so will complete a daily diary of symptoms if they do become unwell to give a more detailed understanding of the course of each illness. A sample of patients and healthcare practitioners will be asked to take part in a telephone interview about their experiences of taking part in the trial.

## Study flow chart

**Practices identified:**

- Approximately 200 GP practices nationally

Patient identification

**GP practice recruitment**

- Practice database searches and letter of invitation and PIS sent by practice
- Opportunistic recruitment via consultations with GP or practice nurse
- Study advertisement posters within practices

**Consent and Screening**

- Patients will visit IMMUNE DEFENCE study website and register
- Online consent and screening to assess eligibility.

**Baseline measures and Randomisation:**

- Baseline questionnaires completed online
- Randomisation stratified into three strata I) risk factors alone ii) risk factors plus recurrent RTIs iii) recurrent RTIs alone

| **Gel-based Nasal Spray** N=3750 | **Saline Nasal Spray** *N* = 3750 | **Getting Active and Managing Stress** N=3750 | **Usual care plus brief advice** N=3750 |
|---|---|---|---|

**Patient illness diaries (during infections) – paper based - optional**
**Monthly questionnaires – online**

**Primary outcome assessment online (6 months)**
Qualitative interviews with purposively sampled participants also conducted during first six months

**Outcome assessment online (12 months):**
(plus medical notes review)

# 1 Background

## 1.1 Introduction: 'Non-pandemic' winters

Most people suffer a respiratory tract infection (RTI) each year and they are the most common reason for sickness absences (34 million days; ONS, 2016). Both upper respiratory infections (URTI – colds, sore throat, sinusitis) and lower respiratory infections (coughs, chest infection bronchitis, pneumonia) are caused in most cases by viruses – commonly rhinoviruses, coronaviruses, influenza viruses and Respiratory Syncytial Virus (RSV) [1]. Whilst RTIs are such a common health problem, individuals with recurrent infections are a higher initial priority: they have more days of illness, more severe illness, worse quality of life and higher work absence.[2-4]

The majority of patients attending their GP with RTIs are prescribed antibiotics[5 6] and primary care antibiotic use is strongly related to the threat of antibiotic resistance[7]. Prescribing antibiotics 'medicalises' illness[8 9]: those with recurrent infections are more likely to return (IRR 2.55) for further antibiotics[8], resulting in an estimated 35% of all new antibiotic prescriptions for LRTI annually, and in maintaining a longer cycle of re-attendance and re-prescribing. The DESCARTE (sore throat) cohort study (n=11,950)[10] reveals very similar findings.

## 1.2 COVID-19 pandemic

The pandemic is caused by a coronavirus SARS-CoV-2 which is closely related to both the virus which caused the SARS outbreak in 2003 and is a cousin to the coronaviruses which cause 'normal' coughs and colds. The range of 'normal' respiratory viruses have also been circulating during the pandemic – and most patients with viral infections presenting with 'COVID-like' infections do not have COVID-19 related illness even when they are admitted to hospital[11], and those who do have confirmed COVID-19 illness also commonly have co-infections with other viruses[12].

## 1.3 Simple non-specific measures to modify the physical environment of respiratory viruses: the role of temperature and pH in lowering the size of the viral inoculum

[redacted]

## 1.4 Empirical evidence of reducing incidence and symptoms of RTIs in humans through anti-viral nose sprays in non-pandemic years.

[redacted]

[redacted]

[redacted]

As part of the development phase of the Programme we have developed intervention materials to encourage the uptake and use of nasal sprays for both prevention and also early in the course of the RTI.

## 1.5 Reducing illness episodes through physical activity and stress management

Physical activity and stress management both improve immune function and have empirical evidence of benefit in reducing illness episodes, so addressing both could plausibly help both the severity and duration of illness.

A recent Cochrane review[26] of the effect of physical activity on reducing illness episodes suggests promising effects for recurrence (risk ratio 0.76 (95% CI 0.57 to 1.01)) and symptom days, but most were small trials of mostly low quality. One high quality small US trial documented a reduction in illness of more than 3 days[27] and these results are supported by a more recent trial[28]. There are similar estimates from a cohort study among 1002 adults[29]. Exercise-related symptoms and restarting activity following illness are particular problems for those with recurrent illness, so simple generic advice is unlikely to work.

Perceived stress[30], negative emotion[31], and poor social support[32] predict subsequent illness, viral shedding, cytokine activity, as well as adverse mucosal defence and pathogenicity[33 34]. Mindfulness can reduce stress and negative emotions[35]. A small US trial of an 8 week course documented a reduction of 3-4 illness days compared with controls[27] and the most recent trial[28] by the same group showed a reduction of 1 day. However, the US trials involved either rather intensive supervised exercise (8 sessions) or similarly intensive supervised mindfulness courses (again 8 sessions), each session being at least 2.5 hours.

We have developed a complex intervention incorporating both physical activity and stress management (plausibly generating additive effects) which is both pragmatic and can be implemented in routine NHS settings. Based on the efficiencies of digital platforms for behaviour

change, we have developed a brief digital intervention requiring minimal support, which is both accessible to patients and efficient for healthcare delivery.  The Lifestyle  intervention is based on the evidence-based modules of our POWeR+[36] and CLASP[37] ‘Getting Active’ interventions, underpinned by self-determination theory[38-40]; and acceptable and accessible on-line stress-management modules through our ‘Healthy Paths’ intervention[41].

This intervention is likely to be as relevant during the COVID pandemic as in normal seasons – particularly as levels of anxiety and stress will be higher, and as people return to work levels of physical activity that have been strongly encouraged to date during the pandemic need to remain a priority.

## 1.6 Progress of existing grant

The work to date has been funded as part of the NIHR Recur Programme. We are at the stage of being close to finishing the development of the online platform and the patient facing materials. We had planned a large feasibility trial this year in the 2020-21 winter season, and a full trial in a ‘normal’ year in the 2021-2023 winter seasons. However, the stage of development this year and our previous experience of running large similar trials suggests we could  do a very much larger full trial starting in the 2020-2021 winter season. This will be able to provide information not only about whether such interventions are likely to work in a pandemic but also in more ‘normal’ years subsequently – whatever the new ‘normal’ turns out to be.

## 1.7 Summary

We propose to evaluate the impact of a nasal spray that buffers pH and uses a polymer, a saline nasal spray, and a complex intervention of physical activity and stress management compared with usual care on the health outcomes for patients at risk of serious illness, including those with recurrent RTIs in primary care.

# 2 Aims and objectives

## 2.1 Aim.

This study will estimate the effectiveness and cost-effectiveness of commonly available nasal sprays and a brief physical activity and stress management intervention in preventing and reducing the incidence, severity and duration of RTIs among patient at risk of serious infection in the COVID pandemic.

## 2.2 Objectives:

### 2.2.1 Primary objective

To assess whether three trial interventions (1)a microgel nasal spray 2) a nasal saline spray, or 3) support for physical activity and stress management reduce the duration of illness days due to respiratory tract infections (RTIs) among at-risk individuals when compared to usual care

### 2.2.2 Secondary objectives

To assess whether three trial interventions (1) a microgel nasal spray, 2) saline nasal spray, or 3) support for physical activity and stress management reduce:

i. the incidence of all respiratory tract infections
ii. health service contacts
iii. hospital admissions
iv. health service resource use (and to estimate cost-effectiveness of each intervention)
v. antibiotic use
vi. the incidence of COVID-like infections (during winters when COVID is circulating)

To evaluate patient engagement with the interventions by exploring patients' experiences of the different interventions to understand why different patients did or didn't engage with the treatment/intervention, and what might affect future engagement.

# 3 Methods

## 3.1 Design

A mixed method, open, randomised, 4-arm trial evaluating i) usual care plus brief advice, ii) a microgel nasal spray, iiI) saline nasal spray, and iv) Lifestyle intervention, for the prevention of recurrent RTIs.

We are interested in assessing the impact both overall and in at risk subgroups of patients defined by whether they a) have comorbidity risk factors for infections/adverse outcome (e.g. immune compromise, older age, serious comorbidities) and/or b) whether they have had recurrent infections in the past (the PRIMIT trial demonstrated that those having 3 or more infections in a normal year were three times more likely to contact an infection in subsequent winter seasons). Thus we wish to provide estimates for three strata of patients – those with risk factors, those with recurrent infections, and those with recurrent infections and risk factors.

## 3.2 Participants

Participants will be adults aged 18 or over

### 3.2.1 Inclusion criteria

**All participants**

1) Patients aged ≥18 years with a risk factor:
   a) Known weakened immune system due to a serious illness or medication (e.g. chemotherapy);
   b) Known heart disease;
   c) Known asthma or lung disease;
   d) Known diabetes ;
   e) Known mild hepatic impairment;
   f) Known stroke or neurological problem;
   g) Obesity (BMI≥30)
   h) Patients with >=3 episodes of an RTI in the last year

   **AND**

   Normally experience one or more RTIs per year

2) Patients aged ≥65 (*criterion removed for season 3*)

   **AND**

   Normally experience one or more RTIs per year

The criteria are very similar to the inclusion for the primary care platform trial PRINCIPLE, but with a wider age criterion (younger patients who have serious co-morbidities) are also likely to be at risk, and including those with a history of recurrent infections (the PRIMIT trial demonstrated that those who reported 3 or more infections in the previous year were more than three times more likely to contract infections during the course of the study). Our first season of recruitment of 3360 participants found that a third did not report a RTI in the 12 months before COVID and more than half did not report an RTI in the year before joining the study. Qualitative interviews have found that some participants are wanting to help research but may not engage in the infection prevention behaviours as they don't consider themselves particularly at risk of catching infections. As we move towards a population level vaccination and more 'normal' winters for respiratory infections (especially in recruitment season 3), we feel that it would be appropriate to tighten our inclusion criteria to include participants who report at least one infection in a 'normal' year. This will give a better chance of engagement with our interventions, includes participants who are likely to be more at risk of getting an infection in a normal winter, and increase likelihood of demonstrating an effect of reducing number or severity of infections.

We have reached the end of season 2 in a strong position with 10,013 participants recruited into the trial. However, 3223 (32%) are participants who report have recurrent infections (defined as 3+ infections in a normal year). We would like to increase recruitment to our 2 recurrent infection strata (+/- a risk factor). We have found that healthy over 65yrs participants (inclusion criteria 2) are less likely to report recurrent infections than those reporting a co-morbidity/risk factor (inclusion criteria 1). We therefore propose to exclude healthy over 65s for season 3. This will have the potential to fill our recurrent infection strata, whilst reducing the number of practices/invitation letters that are needed to reach our targets.

3) Have access to the internet

### 3.2.2 Exclusion criteria

- Terminal illness/palliative care;
- living with dementia
- living in residential care
- pregnancy or breast-feeding;
- pituitary adenoma/resection
- regular use of [redacted] or similar nasal sprays for respiratory infection control in the last 6 months
- allergy to nasal sprays;
- Living in the same household as another participant
- Previously involved in RECUR development work

## 3.3 Sample size

Based on the prior research we anticipate a reduction in both the incidence of infections, their severity, and their duration.

**Primary outcome: number of days of illness in total due to RTIs.** We anticipate that even with the easing of lockdown there will be considerable use of social distancing. It is likely that these behaviours could continue during the winter seasons in subsequent years when the COVID pandemic is over, since patients at risk from infections will realise that these behaviours have provided protection from infections. This means that we should anticipate that the infection rates for both the range of winter viruses including COVID-19 while it is circulating will be substantially lower than in normal years, and that this is likely to continue over the next 2-3 years. In the target population we assume it is not likely to be lower than 15%-20% over a 6 month winter/spring season. We wish to have the most power for this outcome since we wish to compare not only each group with control but each intervention group with each other, and therefore for this outcome we will allow for multiple testing and an alpha of 0.01. Using the data from PRIMIT [21] , to detect a 1 day difference among individuals having an infection (hazard ratio 1.2) for alpha of 0.01 and 90% power requires 147 individuals per group, and allowing for at least 15% of individuals to contract an infection during a 6 month winter/spring period 980 individuals per group are needed and assuming 4 groups and 80% follow-up (which we achieved using similar methods to the PRIMIT trial), then 4900 individuals are needed. A 1 day difference is smaller than the difference found for both saline and [redacted] in the previous trial[22], and the minimum required in discussion with our PPI collaborators.

Thus 5000 is the minimum sample required but as in the original application preferably we wish to estimate these outcomes in each stratum (i.e. 14,700 patients), and so will aim for 15,000 participants in total, which is feasible since the method of recruitment is as with PRIMIT by mailed invitation. During the first season, which will be during the second winter of the COVID pandemic, we anticipate we will quite possibly be able to recruit 5000 patients. If this is the case, then even during the first season, although we will not be powered for each stratum, there will be adequate power in the whole trial sample to provide useful information about the role of these interventions during a future pandemic (as the previous PRIMIT trial has informed the importance of handwashing in the current pandemic).

**Occurrence of infections.** We will have less power for this outcome, but if our primary aim for this outcome is to compare each intervention with control, using the arguments of Cook and Farewell, since each analysis of intervention versus control is independent, this should not require a

conservative Bonferroni correction[42], and we can use an alpha of 0.05. Using these assumptions the above sample size will provide more than 80% power to estimate a 25% reduction in the incidence of infections from 20% to 15% in each stratum [redacted] and more than 90% power if the incidence of infections is 15% using all strata combined.

# 4 Interventions

**Active treatment arms**

Patients in all three active treatment arms will be shown a series of webpages explaining what RTIs are and summarising their impact on health, work and social life. The webpages then proceed to build a rationale for how either saline or [redacted] nasal sprays or lifestyle change may help reduce the number of RTIs experienced (& their length and symptom severity). Participants are then directed to digital interventions supporting them to either use [redacted] nasal spray, saline nasal spray, or to make lifestyle changes (physical activity and stress management).

## i. [redacted] nasal spray [redacted]

Participants in this arm access a digital intervention that provides explanations and instructions on how to use the spray, including a demonstration video. Concerns (e.g. about side effects) are addressed through FAQ style content and tips to help people to remember to use and to adopt the correct technique are included. This information is duplicated in a paper booklet that is then sent out to participants along with bottles of the spray. The study team will receive an automated email notification from the trial website when a patient is randomised to [redacted]. Two bottles of [redacted] (masked with study labels) together with the printed instruction booklet will be mailed to the participant in the first instance and further bottles will be provided on request to the study team. [redacted]

Participants will be asked to use VDF nasal spray 12 sprays per day in each nostril (2 sprays 6 times per day) during the prodrome (e.g. malaise, low grade fever, appetite loss) or in the earliest part of the illness when respiratory symptoms develop and to continue using the spray until symptom-free for 1 day.

Additionally, participants will be asked to use VDF nasal spray preventatively following significant exposure (e.g. < 2 metres from someone outside their household unit for >=5 minutes that day, following visits to the supermarket or other shops lasting more than 5 minutes). Participants will be asked to use the spray immediately following exposure (to inactivate most virus at the time), one hour later, and again at night to limit the start of viral replication.

### ii. Saline nasal spray

Participants in the saline spray arm will be given access to a website that provides explanations and instructions on how to use the spray, including a demonstration video. Concerns (e.g. about side effects) are addressed through FAQ-style content and tips to help people to remember to use and to adopt the correct technique are included. This information is duplicated in a paper booklet that is then sent out to participants along with bottles of the spray. The study team will receive an automated email notification from the trial website when a patient is randomised to Saline Nasal Sprays. One bottle of Saline spray (30ml), together with the printed instruction booklet, will be mailed to the participant in the first instance and further bottles will be provided on request to the study team.

Saline nasal spray [redacted]
The manufacturer's labels will be removed and replaced by generic study labels.

Participants will be asked to use the saline nasal spray 12 sprays per day in each nostril (2 sprays, 6 times per day) during the prodrome (e.g. malaise, low grade fever, appetite loss) or in the earliest part of the illness when respiratory symptoms develop, and to continue using until symptom-free for 1 day.

Additionally, participants will be asked to use Saline nasal spray preventatively following significant exposure (e.g. < 2 metres from someone outside their household unit for >=5 minutes that day, following visits to the supermarket or other shops lasting more than 5 minutes). Participants will be asked to use the spray immediately following exposure, one hour later, and again at night.

In a small number of practices we may evaluate the feasibility of patients collecting the nasal sprays ([redacted] and Saline) from their GP surgery or local pharmacy.

### iii. Lifestyle intervention.

Lifestyle Intervention: Participants randomised to the Lifestyle intervention will be given access to the two intervention modules online to support a) physical activity, "Getting Active" and b) Stress reduction, "Healthy Paths through Stress". Participants are encouraged to try both interventions and will have access to them at all times. These are complex interventions which could plausibly generate additive effects of the individual components. Both interventions were adapted from established digital interventions.

**Physical Activity**

The 'Getting Active' digital intervention was developed and evaluated in previous research including POWeR+, an intervention to manage obesity in primary care and CLASP, an intervention to support cancer survivors to make lifestyle changes to boost quality of life.

‘Getting Active’ has been optimised for an RTI context, through iterative qualitative think-aloud interviews and retrospective interviews with people who experience recurrent RTIs, using the person-based approach[43]. It builds motivation for physical activity, makes suggestions for types of physical activity and increases people’s confidence in being able to do so, supporting them to set and monitor their own goals, count and record steps, and receive automated tailored feedback and support. It has content addressing concerns about getting active and overcoming barriers and uses user’s stories to model achieving physical activity in the context of busy lives, low confidence, lack of money/facilities and chronic health conditions. Our participants are likely to be a heterogenous group in terms of age and health status but we expect a significant proportion to be older, and have chronic health problems (e.g. COPD, asthma) in addition to recurrent RTIs. As such, Getting Active does not prescribe a specific type and amount/intensity of activity but supports people to safely extend what they are currently doing. It emphasises lifestyle activity (walking, gardening) and activity that is matched to the person’s ability and personal preferences. It contains material to support medium- or longer-term goals of achieving/maintaining NHS recommendations for physical activity (e.g. 150mins moderate activity per week, etc). “Getting Active” sends automated emails encouraging and supporting participants to engage with the intervention and overcome barriers to physical activity. A step counter will be sent out by post to each participant at the recruitment stage.

**Stress management**

“Healthy Paths through Stress” was initially developed as a digital intervention for stress/emotional distress for patients in primary care[41] but has also been used to manage distress in cancer survivors (NIHR-funded Cancer: Life Affirming Survivorship support in Primary care programme – CLASP)It has had both technical updates and optimisation for the current context via think-alouds and retrospective interviews as described above. In Healthy Paths participants can explore a range of evidence-based techniques and read rationales and instructions for trying them. These are drawn from behavioural activation (pleasant activity scheduling, sleep hygiene) and/or mindfulness-based approaches (e.g. 3-minute breathing space, self-compassion exercise). Participants can select those they find most helpful. They will be asked to engage with these cognitive and behavioural strategies on a regular basis and particularly when encountering stressful life events.

### iv. Usual care with brief advice:

Usual care will comprise a brief page of advice about managing respiratory illnesses, based upon NHS current advice. The usual care group will be asked not to use any over-the-counter nasal sprays during the study period.

# 5 Recruitment

## 5.1 Practice recruitment

GP Surgeries will be invited to take part via the Clinical Research Networks. Wherever possible, practices will be selected to represent a range of socio-demographic factors, including those from urban and rural settings, large and small practices, and areas of high and low social deprivation. Assuming a 10% uptake in our target population we will need 200 surgeries to participate in mailouts.

## 5.2 Patient recruitment

#### 5.2.1.1 Targeted invitation

We anticipate that this method of recruitment will provide the vast majority of patients as it did in the PRIMIT trial. Practices will be provided with executable files to identify potentially eligible patients from the clinical record system. The lists will then be visually checked against the exclusion criteria by a practice GP to ensure suitability of patients to receive an invitation. A secure mail service (Docmail) will be used to send invitation packs containing an invitation letter, participant information sheet, and a 'How to Take Part' sheet providing instructions about how to participate. Invitation packs may otherwise be sent out directly by the practice rather than via Docmail. Each pack will be pre-numbered with a unique participant code which is required to sign up to the study website. Patients who do not wish to take part can provide feedback and reasons for non-participation by email or, anonymously, through a link to a brief questionnaire on the Immune Defence website.

**Season 3:**

We are seeking approval from the REC to amend our recruitment materials for season 3. Currently patients receive an invitation pack containing the invitation letter, participant information sheet and 'how to take part' sheet. This makes a large 8-page invitation pack which is both costly and can be overwhelming/inaccessible for participants. We propose to mail out a brief study information sheet and direct participants to the detailed online information sheet. Our rationale for this is three-fold. Firstly, participants who are invited by text messaging already access the participant information sheet online and are able to print out if needed. We will also offer to post a copy of the information sheet to participants if requested. Immune Defence is an online study and participants do need access to to a computer or mobile device to take part, so we don't think accessing the participant information in this way will be a barrier to being fulling informed about the study. Secondly, qualitative feedback from participants suggests that such a large mailpack can be quite off-putting. We are also working with our PPI panel to widen participation to more socially and ethnically diverse groups, and the panel feels that a more accessible invitation pack may assist with this. Thirdly, there is a cost consideration. Each invitation pack costs £1.12 (we sent >250,000 invitation packs last season). Reducing the size of the pack will reduce the individual cost to £0.90, potentially saving >£50,000 this season for a similar mailout. Changing the mailpack will allow us to invite more participants with the funding available. Without this we may be at risk of being unable to reach our recruitment targets (response rates are lower this season compared to the first two seasons during the pandemic).

In addition to this we would like to extend the use text message invitations to the targeted patient group. This has the potential to greatly reduce recruitment costs whilst ensuring that we reach our recruitment targets. As in section 5.2.1.2, practices will conduct a search of their records using executable files provided by the study team to identify potential participants and send a standard text message with a link to the study details. Participants will be able to express interest in the study by completing an online form or by contacting the study team directly. Participants will then receive a study pack (by email) and enter the study in the normal way. Sites will either use text message or postal invites (not both methods in the same season).

In order to raise awareness of the study in GP practices, particularly in those in regions of higher ethnic diversity and lower sociodemographic status, we will ask practices to display their recruitment poster (as described in section 5.2.1.3). Patients will be asked to consider joining the study if they

receive a postal or text invite, and asked to contact the study team if they need any further information.

#### 5.2.1.2 General invitation to wider population

Consultations for respiratory tract infections, some serious comorbidities, and reasons for antibiotic prescriptions are not necessarily recorded in medical records in a consistent manner. Therefore, it is possible that there will be more patients who would be eligible for the study who are not identified through targeted invitation. If the yield from targeted invitation is low, which seems unlikely in the current climate of anxiety during the pandemic, we will consider conducting a wider mailout to patients 18+ in a number of GP practices. We have successfully used this method in the PRIMIT study.

Season 3: whilst targeted invitation has been very successful overall in recruiting participants to the trial, it has been more difficult to recruit people under 65yrs with no comorbidities/risk factors plus recurrent infections. In order to increase numbers in this strata we would like to trial the use of text message invitations to a wider cohort of under 65yrs with no comorbidities. Interested participants who self-report 3+ infections in a normal year and have consulted their GP in the last 12 months about a RTI will be able to sign up for the study. Text message invites from GP practices have been very successful in recruitment to the national PRINCIPLE and PANORAMIC trials. We will trial this method in a number of practices who use text messaging service to communicate with their patients (text messaging is commonly used as consultation reminders, flu and Covid vaccine reminders etc). Practices will conduct a search of their records using executable files provided by the study team to identify potential participants and send a standard text message. Interested participants will visit the study website hosted by the University of Southampton for full details. Eligible participants (3 or more infections plus a consultation with the GP) will be able to express interest in the study by completing an online form or by contacting the study team directly. Participants will then receive a study pack (by post or email) and enter the study in the normal way.

#### 5.2.1.3 Opportunistic recruitment

Patients will be approached by GPs and practice nurses/nurse practitioners in their normal consultation, which for many patients during the COVID period is likely to be by telephone or video consultation. GPs/nurses will check the patient records for eligibility. Interested patients will be sent an information pack containing an invitation letter, participant information sheet, and a 'How to Take Part' sheet giving instructions about how to participate. Each pack will be pre-numbered with a participant code to sign up to the study website. Practice staff will keep anonymised screening logs with details about patients they have approached for the study.

We will include posters in the GP surgery and interested patients will be asked to speak to their GP or practice nurse, who will assess eligibility for the study. Eligible patients will receive an information pack as detailed above. Give the pressures on primary care we anticipate that most recruitment will be via postal invitation.

#### 5.2.1.4 Community recruitment/outreach

Our PPI panel members have been discussing ways of raising awareness of the Immune Defence study to wider community groups. We are planning a number of outreach sessions at local church

and community organisations to talk about research in general, and the Immune Defence study. We would like to provide brief information about the study to interested members of the public. Any person who is interested in finding out more about the study will then be sent a participant invitation sheet by email or post after the meeting and can enter the study in the usual way if they are eligible. These participants are not therefore recruited by their GP surgery so baseline reporting of risk factors will be by self-report. We think this is an important way of recruiting a more diverse group of participants, and also has the potential of building links with community groups for future trials.

# 6 Trial procedures

Interested participants will visit the Immune Defence Trial website and sign up with the unique identifier from their invitation pack. Participants will then enter their username (email address) and choose a password. Study team contact details will be available so that participants can get in touch with any questions.

## 6.1 Informed consent and screening

Patients will be asked to give their consent online prior to completing the screening questions. Screening questions will ask for details about number of RTIs in the last year and any exclusion criteria.

Patients who are not eligible for the study will be thanked for their interest and given the link to a brief NHS advice page about managing respiratory illnesses.

## 6.2 Baseline measures

Patients who meet the screening criteria will be asked to complete the baseline measures online.

At baseline patients will complete the following:

- Name, address, contact telephone number.
- Gender, age; marital status; years of education; ethnicity; height and weight; smoking status; number of RTIs in last year; total days with symptoms, days moderately bad or worse, days lost to work/other activities; visits to the doctor for RTIs in the past year; number of antibiotic prescriptions for RTIs in the past year; COVID symptoms in the last 12 months; results of COVID testing (PCR or antibody) in the last 12 months; other health problems; influenza vaccine in current season; COVID vaccine in the last 12 months; number of children <16years in the household; number of household members;
- Baseline questionnaires including Beliefs in antibiotics, International Physical Activity Questionnaire/Domain-Specific Sitting, perceived stress scale, PHQ-8, GAD-7, and EQ5D-5L

## 6.3 Randomisation procedures

Following completion of the baseline measures, participants will be randomised by the Immune Defence website to one of the 4 study arms:

The randomisation process (1:1:1:1). for this trial will be fully automated. The intervention and data collection software generates a randomisation sequence and a computer algorithm will block randomise participants to the trial groups. As the randomisation is automated, the randomisation sequence will be concealed from the trial team.

Patients will be stratified on the basis of being in a higher risk group (over 65 and/or having comorbid condition) and whether or not they have recurrent RTIs (≥3 in the last year) to three strata:

1) risk factors (comorbidities and/or over 65) plus recurrent infections

2) risk factors (comorbidities and/or over 65) alone

3) recurrent infections alone.

## 6.4 Blinding

Full blinding of study participants to their intervention group is not possible. However, to reduce possible contamination through the potential wider use of [redacted] during the current pandemic situation, we propose masking the nasal sprays to their content, by removing the manufacturers labels, and adding generic study labels (or overlabelling).

Staff responsible for data entry and analysis will be blind to the study group.

## 6.5 Outcome data collection

### 6.5.1 Outcome measures

**<u>Table 1: Outcome and process measures</u>**

| Measures | Screening | Baseline | Illness diaries | 4 weeks | 8 weeks | 12 weeks | 16 weeks | 20 weeks | 6 months | 28 weeks | 32 weeks | 36 weeks | 40 weeks | 44 weeks | 48 weeks | 12 months |
|---|---|---|---|---|---|---|---|---|---|---|---|---|---|---|---|---|
| **Screening** | x | | | | | | | | | | | | | | | |
| **Respiratory illnesses:** | | | | | | | | | | | | | | | | |
| Number and type of infection | | x | x | x | x | x | x | x | x | x | x | x | x | x | x | x |
| Checklist of symptoms if infection occurred | | | x | x | x | x | x | x | x | x | x | x | x | x | x | X |
| Days with infection | | x | x | x | x | x | x | x | x | x | x | x | x | x | x | x |
| Days with mod /severe infection | | x | x | x | x | x | x | x | x | x | x | x | x | x | x | x |
| Symptom severity | | | x | | | | | | | | | | | | | |
| Days off work /normal activities | | x | x | x | x | x | x | x | x | x | x | x | x | x | x | x |
| Appointments with HCP | | x | x | x | x | x | x | x | x | x | x | x | x | x | x | x |
| If tested for COVID and the result | | x | | x | x | x | x | x | x | x | x | x | x | x | x | x |
| **COVID vaccine** | | x | | | | x | | | x | | | | | | | x |
| **Vitamin D** | | | | | | | | | x | | | | | | | x |
| Courses of antibiotics | | x | x | x | x | x | x | x | x | x | x | x | x | x | x | x |
| **Severity/other health problems** | | x | | | | | | | | | | | | | | |
| **Psychological Process measures** | | x | | x | | | | | x | | | | | | | x |
| **Adherence to treatments/intervention** | | | x | | | | | | x | | | | | | | x |
| **Beliefs in antibiotics** | | x | | | | | | | x | | | | | | | x |
| **Intention to consult** | | x | | | | | | | x | | | | | | | x |
| **Demographics** | | X | | | | | | | | | | | | | | |
| **IPAQ** | | x | | | | | | | x | | | | | | | x |

| | | | | | | | | | | | | | | | | |
|---|---|---|---|---|---|---|---|---|---|---|---|---|---|---|---|---|
| **Domain specific sitting** | | x | | | | | | | x | | | | | | | x |
| **PHQ-8** | | x | | | | | | | x | | | | | | | X |
| **GAD-7** | | x | | | | | | | x | | | | | | | X |
| **EQ5D** | | X | x | | | x | | | x | | | | | | | X |
| **Out of pocket expenses** | | | | | | x | | | x | | | | | | | x |
| **Cold avoidance behaviours** | | x | | | | x | | | x | | | | | | | x |
| **Use of nasal sprays** | | x | | | | x | | | x | | | | | | | x |
| **Adverse events** | | | | | | x | | | x | | | | | | | x |

### 6.5.2 Primary outcome measure

The primary outcome will be the number of days of illness due to respiratory tract infections (RTIs) in total due to respiratory tract infections (RTIs) over 6 months (based on the report at 6 months and from the monthly proformas).

### 6.5.3 Secondary outcome measures

Patient reports in monthly proformas and after 6 and 12 months:

- The number of days with symptoms of RTIs rated moderately bad or worse
- The number of days where work/normal activities were impaired
- incidence of infection with RTIs
- whether contact with the health service was needed;
- whether antibiotic was taken

Beliefs about antibiotics and intention to consult: Patient beliefs in the efficacy of antibiotics and their intention to consult in the future will be measured using a 4-point Likert scale (*very likely, moderately likely, slightly likely, not at all*) at baseline, 6 and 12 months. We have shown these outcomes to be responsive to interventions that provide alternatives to using antibiotics[9 44 45]

Physical activity: We will measure patients' physical activity using the Short form International Physical Activity questionnaire[46] and sitting behaviours questionnaire[47] at baseline, 6 and 12 months. However, questionnaires alone are unlikely to be a significant prompt for behaviour change, and may be helpful at documenting change at a group level but not very useful at an individual level. Therefore, we propose using activity monitors in the Lifestyle intervention group to document change, and activity monitors will also be part of the intervention and will help change at an individual level.

Work absence: Absence from work will be recorded from self-report monthly.

Mental health: Patient's mental health will be assessed using The Perceived Stress Scale[48], PHQ-8[49] and GAD-7[50] (Generalised Anxiety Disorder scale)at baseline, 6 and 12 months since the Lifestyle intervention might plausibly improve mental health outcomes and presence of mental health problems/distress may predict engagement with the health paths part of the lifestyle intervention.

Quality of life (QOL): Patient QOL will be measured using EQ-5d-5L[51] at baseline, 3, 6 and 12 months which we anticipate will capture impact on both RTI and non RTI-related quality of life. However, QOL for RTIs is part-driven by inter-current illness. Therefore we will collect EQ-5d-5L[51]data as part of the symptom diaries which will supplement the main EQ-5d-5L[51] data

NHS contacts: The number of contacts with the NHS will be measured by self-report in the monthly proformas and at 3, 6 and 12 months, and by retrospective notes review. Health service contacts include NHS 111, primary care, A&E and out of hours services. We hypothesize that Lifestyle intervention and [redacted] will reduce the number of health service contacts by reducing the number of RTI episodes.

Out-of-pocket spending will be collected through patient self-report at 6 and 12 months. We will collect data mainly on RTI related medication and NHS service use since these elements of resource use are by far the most likely to be affected.

Side effects: Side effects to the nasal sprays will be recorded in the monthly questionnaires and at 3, 6 and 12 months (e.g. nasal irritation, stinging, nose bleeds).

Engagement with the trial interventions: We will explore patients' experiences of the trial interventions to understand why different patients did or didn't engage with the treatment/intervention, and what might affect future engagement, through quantitative psychological process measures and a qualitative process evaluation.

Engagement with all intervention arms will be automatically collected through usage data from the website. This data includes when, how often, and for how long participants logged on to the intervention, which content was accessed, for how long and in which order and engagement with specific tools or features (e.g. goal setting)

### 6.5.4 Methods of data collection

Each month patients will receive an automated email asking them to complete online the brief monthly measures including whether an RTI occurred, a checklist of what the symptoms were during the first week (which will enable classification of whether the RTI is a COVID-like illness), whether a COVID test was performed (participants will be asked to attend for a routine test available in the NHS should they have COVID-like illness), and whether the test was positive, whether they had further care (e.g. saw the GP, were admitted to hospital) number of days with symptoms and symptom severity in the previous month. Participants will receive an email reminder after 1 week if the questionnaires are not completed. No further reminders will be sent if the measures are not completed. We have shown previously that patients can reliably remember the duration of infections over a matter of weeks[52] and also shown that over several months when patients are aware that they will be asked about infections regularly there is perhaps surprising agreement

between the estimates based on monthly measures and the estimates based on measures reported at 4-6 months.[21]

### 6 and 12 month measures

Participants will complete additional follow-up measures at 6 and 12 months. As with the monthly measures described above, participants will receive an automated email asking for them to be completed and a reminder email after 1 week

If, after two weeks, participants have not completed the online measures, paper-based questionnaires will be sent in the post with a freepost envelope for returning to the study team. If after 3-4weeks there is no response from the participant, research staff will make contact with participant by telephone to request limited responses over the phone (number of RTIs/antibiotic use). A brief text will be sent ahead of the telephone contact to let participants know when a researcher will be calling.

If at any stage the participant indicates that they would not be willing to complete any further measures, no further contact will be made.

To maximise recruitment we have kept the basic outcomes very simple – to minimise any barrier to recruitment. Nevertheless, we wish to gain further information for those participants willing to do so – both more information on the pattern of illness and quality of life using a daily diary.

### Optional Illness diaries

We will ask patients at the baseline assessment if they would be willing to complete a symptom diary should they develop an RTI during the study period. We will collect details of symptoms and symptom severity, medications taken, health care contacts, and EQ5D. Additionally, participants in the saline spray and [redacted] groups will be asked to record adherence to their nasal sprays.

Initially paper-based diaries will be used, but online data capture with Qualtrics or similar secure platform will be investigated. Paper diaries will be sent by post to the participant following randomisation, and participants will be asked to post the diary back to the study team in a freepost envelope.

### End of the study

At the end of the study, participants will be thanked for their participation in the study and offered a summary of the results when available. Participants in the usual care arms will be offered access to the Lifestyle intervention for a brief period after the end of the study. Participants in the nasal spray groups will be able to request further supplies of the masked nasal spray after they have completed the 12 month study period. Whilst both nasal sprays in the study are available to purchase over the counter in pharmacies and supermarkets, we would like to ensure participants remain masked to the trial nasal sprays until the study completes in 2024 (to preserve blinding).

## 6.6 Nested qualitative interviews

A purposeful sample of up to 100 patient participants (or until data saturation is reached) will be invited to take part in a semi-structured interview with a researcher experienced in qualitative research methods from different time points throughout the trial. Patients who have consented to be contacted for interview will be emailed/posted an invitation letter and participant information sheet. Interested participants will complete a consent form (on paper or online) or give verbal consent for interview over the telephone with the researcher. The sample will include patients from

each of the four intervention arms; from different GP practices covering a range of social deprivation levels; from the general and more severe sub-group; and with a range of ages and gender. We will also include some participants who didn't engage well with the study, including low intervention usage and low questionnaire completion. We will explore participant experiences of the intervention and the trial processes and procedures, identify barriers and enablers to intervention engagement, perceived benefits of the intervention, and trial retention. Each interview will last approximately 60 minutes and take place either face to face (at the participants' home or at the University) or remotely by telephone/Microsoft Teams, and will be guided using a topic guide developed by the study team. We will offer participants a £10 gift voucher to thank them for their participation in an interview.

We will also invite a purposely varied sample of staff (up to 40 participants; or until data saturation is reached) from a range of participating GP practices including GPs, practice nurses and practice managers to take part in a focus group or semi-structured telephone/Microsoft Teams interview. The choice of focus group or interview will be based on what is most convenient for practice staff. An invitation letter/email and participant information sheet will be emailed to the participating practice staff, interested staff will contact the study team at University of Southampton. We will explore experiences of identifying and recruiting patients to the study and perceptions of trial procedures and the interventions. Interviews will take approximately 30-60 mins. If staff are unavailable, written feedback will be sought.

## 6.7 Recording and reporting adverse events

### 6.7.1 Definition of non-serious Adverse Event (AE)/Adverse Device Effect (ADE)

For this study, a non-serious adverse event (AE) is defined as any untoward medical occurrence during the study period which is not defined as serious (see 6.7.2) and which is related to any of the trial interventions. Participants in all treatment arms will be asked to record any adverse events associated with the use of the nasal sprays ([redacted] and Saline) e.g. nasal irritation, stinging andnose bleeds,as well as events associated with exercise such as falls and sprains.. The trial team will also make a record of any AEs/ADEs reported during administrative contact with participants, eg, if the participant seeks advice or wishes to withdraw due to an adverse event. No other adverse events will be routinely recorded.

### 6.7.2 Definition of serious adverse event

This study shall adhere to the EU Medical Device Regulation (MDR) Article 2 (58): , where a "Serious Adverse Event" (SAE) is defined as any adverse event that led to:

a) death,

b) serious deterioration in the health of the subject, that resulted in any of the following:

i. life-threatening illness or injury,

ii. permanent impairment of a body structure or a body function,

iii. hospitalisation or prolongation of patient hospitalisation,

iv. medical or surgical intervention to prevent life-threatening illness or injury or permanent impairment to a body structure or a body function,

v. chronic disease,

c) foetal distress, foetal death or a congenital physical or mental impairment or birth defect.

Expectedness

a) **Expected Serious Adverse Events (SAEs)/Serious Adverse Device Effects (SADEs)**

Expected SAEs are those events which can beexpected to occur in the patient/population or as a result of the routine care/treatment of the patient and do not require expedited reporting

A high rate of hospitalisations, new illness diagnoses and deaths are expected in the elderly and high-risk population included in the Immune Defence Study and as such will be considered expected SAEs.. Expected SAEs in this group wouldinclude, but are not limited to, worsening of pre-existing disease, musculoskeletal injuries including falls, cardiovascular events, respiratory illnesses resulting in hospitalisations/deaths, age-related illness/death, etc. These events will be identifieded by monthly patient report and by end of Study report by the GP practice,

Any case of Trigeminal Neuralgia occurring within the nasal spray treatment arms AND which meets the criteria for being a serious event should be recorded as a Serious Adverse Device Effect (SADE) and should be reported. Non-serious Trigeminal Neuralgia occurring within the nasal spray treatment arms may be recorded as an Adverse Device Effect (ADE) but need not be reported.

**b) Suspected Unanticipated Serious Adverse Device Effects**

An adverse event that meets the definition of 'serious' and which is study-related but which could not be considered to be expected as described above, should be classified as a Suspected Unanticipated Serious Adverse Device Effect (SUSADE) and must be reported immediately to the Sponsor and Research Ethics Committee

### Causality

A SADE occurring to a research participant will be reported to the study team at University of Southampton where, in the opinion of the Principal Investigator at site, the event was related to administration of any of the research procedures (use of the trial website intervention 'Immune Defence', or use of the nasal sprays [redacted] or saline nasal spray) , and was an unexpected occurrence. The causality assessment of the event will be undertaken by the Chief Investigator or other medically-qualified member of the trial team.

### Exemptions

- Pre-planned hospitalisation e.g. for pre-existing conditions which have not worsened, elective procedures for a pre-existing condition will not be classed as an SAE unless deemed related to the study.
- Expected SAEs as detailed in section 6.7.2 (a) and SAEs NOT DIRECTLY related to the study as assessed by PI at site do not need to be reported as an SAE.

However, we will ask sites to notify the study team of any unrelated deaths and hospitalisations (except pre-planned) during the study period using a Change of Circumstances form, to ensure that patients are not inappropriately contacted for trial follow-up if deceased or in hospital.

### Reporting

GP Practices will inform the study team at University of Southampton of any SAEs considered to be related to the study immediately but at least within 24 hours of becoming aware of the event occurring.

SAEs should be reported using the study specific SAE Report Form and completed in as much detail as possible and emailed to the study team at university of Southampton at IDstudy@soton.ac.uk, with SAE in the title of the email.

Note that the initial report can be made by phone but this must be followed up as soon as possible with a paper report form.

All SAEs will be reviewed by the chief investigator or delegated medically qualified doctor.

#### Responsibilities for Safety Reporting to REC

The Study Team will notify the appropriate REC if an SAE is considered related to the trial and unexpected within 15 days of the receipt of the report.

#### Follow Up

All SAEs will be followed up until resolved or an end of study criteria is met (e.g. patient withdrew from study).

All SAEs will also be sent to the sponsor, the Trial Steering Committee and Data Monitoring and Ethics Committee.

## 6.8 Discontinuation/study withdrawal

Patients may withdraw from the study at any time without giving a reason. Unless informed otherwise, any data provided up until that point will still be used. If a patient does give a reason for withdrawal, this will be recorded by the study team.

## 6.9 Definition of End of Study

The end of study is the date of the last follow-up of the last participant.

# 7 Analysis

## 7.1 Statistical analysis

IBM® SPSS® software platform, Stata and Excel software will be used to evaluate outcomes.

The primary analysis will be based on those who report at least one infection in a normal year. We will also conduct a sensitivity analysis to include all participants who were included under the broader inclusion criteria ('at-risk' due to COVID) in season one.

The primary time point for analysis will be at 6 months.

- The analysis of the primary outcome and other continuous outcomes will compare all groups.
- For the incidence of infection data the primary analysis will be between each intervention group and usual care, and if any of these demonstrate an intervention is effective then we propose secondary comparisons between groups.

A secondary analysis will use of repeated measures over the year.

The particular regression models used will depend on the data and the patterns of residuals but we anticipate logistic regression models for dichotomous outcomes, negative binomial models for count data, and generalised linear mixed models will be used for continuous variables (all controlling for baseline values; stratification variables and potential confounding variables). ITT analysis with missing data imputed (via chained-equations multiple imputation model) will be the primary analysis, and complete cases as a sensitivity analysis. Secondary analyses will follow a similar modelling approach to the primary analyses. The repeated measures analysis over the one year period will allow for the clustering of observations within participants over time. Estimates will be provided for key subgroups (e.g. those with recurrent infections (>3/year), age >65, the presence and number of serious comorbidities) A full SAP will be drafted prior to analyses being performed. Results will be reported in line with the CONSORT guidelines.

## 7.2 Qualitative process analysis

We will analyse qualitative data using inductive thematic analysis[53]. Qualitative data from practice staff and patients will be analysed separately to suggest improvements to trial procedures and evaluate whether any modifications to the intervention is needed. Qualitative data from patients will also be analysed to inform choice of process and outcome measures for the full trial. NVivo qualitative data management software will be used to facilitate coding and ensure an audit trail of the analysis is maintained. Qualitative findings will be triangulated with the quantitative analyses[54]. We will examine how and why our qualitative findings converge with, complement or contradict the quantitative findings, for example by comparing patient experiences with trial outcomes.

## 7.3 Quantitative process analysis

We will assess reach (uptake; sample characteristics), self-reported adherence[55], predictors of adherence and outcomes (age; gender; education; comorbidities; illness and treatment perceptions; self-efficacy to overcome identified barriers).

The questionnaire data will be used to assess adherence for nasal sprays. Automatic data collection by the digital intervention will assess engagement with the website. We will examine the moderator effects of baseline characteristics (particularly demographics) on engagement with the intervention and outcomes, and the factors likely to mediate engagement (e.g. adherence, beliefs). We will also employ multi-level modelling to investigate how process measures relate to outcomes.

## 7.4 Health economic analysis

The primary analysis will take a societal perspective covering the intervention costs, NHS and personal social service (PSS) and personal expenses (out-of-pocket spending and employment). The outcome will be expressed as incremental cost effectiveness ratios (£/symptom-day averted) and cost-utility (£/QALY- quality adjusted life years).

Resources to provide interventions will be collated and costed via GP records and a patient self-reported questionnaires. It is recognized that conducting a full case notes review for ~15,000 particpants would be a high burden and high cost for both practices and for the trial team. Participants report health resource use using monthly questionnairs, and response rates are 70-75%. We will therefore use patient monthly self-reported health service contacts and antibiotic use as our main dataset for analysis. For non-responders will conduct a brief notes review. Additionally, we will conduct a detailed notes review on a small percentage recruits (5-10%) to check precision of

self-reported data.  This will consultations for RTIs; hospitalisations for RTIs, exacerbatons of COPD/asthma, cardiovascular events, falls; medications for RTIs, asthma medications, cardiovascular meds.

Out-of-pocket spending, time off work, internet use and physical activity will be collected through patient self-report at 3, 6 and 12 months. We will collect data mainly on RTI related medication and NHS service use since these elements of resource use are by far the most likely to be affected. Although the impact on other conditions is likely to be very much less we will also collect data on those conditions that could possibly be affected by the interventions.

All items will be costed using appropriate data (e.g. PSSRU – Personal Social Services Research Unit, NHS reference costs), with time off work costed at national average wage. Resource use will be weighted by its unit cost. Costs for each patient over the study period will be calculated.

Quality of Life (QOL) will be measured by EQ-5D-5L at baseline, 3 months 6 months and 12 months which we anticipate will capture impact on both RTI-related and non-RTI related QOL.

We will use the UK tariff to translate the questionnaire responses to utility scores. QALYs will be estimated by means of area under the curve. The differences for Cost and QALYs between interventions will be estimated using generalised liner mixed models to deal with the hierarchal structure of the data, and adjusted for baseline characteristics. Where appropriate we will estimate incremental cost-effectiveness ratios (ICERs) for comparing different interventions.

Bootstrapping will generate incremental cost effectiveness ratios (ICERs). Cost-effectiveness acceptability curves will be produced to reflect the probability of an intervention being cost-effective at different given willingness-to-pay values per QALY gained. Major assumptions made in the analysis will be tested by means of sensitivity analyses.

# 8 Dissemination plans

We plan to write up this study for publication in a peer reviewed journal and to disseminate results at primary care conferences and to all participants (GP practices and patient participants).  All participants will be sent an accessible summary of the study findings within 6 months of study completion. We will also disseminate our findings to the wider public via our patient collaborators, PPI panels and through social media. Our named PPI collaborators and the PPI panel will be central in leading the strategy for dissemination.

# 9 Patient and public involvement

Our named PPI collaborators (Samantha Richards-Hall; Samantha Beddoe) have full collaborator status, and a role at all stages in the RECUR programme. They have provided input into the development of this protocol, and all materials associated with this study (patient information leaflets, topic guides). They will attend progress meetings and contribute to management decisions regarding development and operationalising the study. They will also contribute to the writing up of outputs (academic papers) associated with this study.

# 10 Ethical issues

## 10.1 Informed consent

All participants will receive information and will have the opportunity to ask questions prior to deciding whether to take part. For the main study, participants will give consent online after signing up to the Immune Defence trial website. For participants taking part in a telephone interview, written consent or verbal consent over the telephone will be given by the patient to the researcher.

## 10.2 Assessment and management of risk

A full risk assessment will be conducted prior to commencement of the study

The topic being investigated relates to respiratory tract infections. It is anticipated that no information will be shared with the research staff that will have safeguarding implications.

The University of Southampton has a lone working policy which will be strictly adhered to at all times to minimise the risks to the researchers.

## 10.3 Research Ethics Committee (REC) and other Regulatory review & reports

Before the start of the study, a favourable opinion will be sought from the HRA (Health Research Authority) and an NHS REC for the study protocol, informed consent forms and other relevant documents.

## 10.4 Indemnity

The University of Southampton Professional Indemnity insurance and/or indemnity will apply to meet the potential legal liability of the sponsor for harm to participants arising from the management of the research.

The University of Southampton Professional Indemnity insurance and/or indemnity will meet the potential legal liability of the sponsor for harm to participants arising from the design of the research.

General Practitioners, who are acting as investigators or collaborators, will have their own insurance and/or indemnity to meet the potential legal liability of any activity being carried out on the study. Evidence of this will be sought where appropriate.

# 11 Data Management, Data Protection, Data Security

## 11.1 Personal Data

Participant personal data will be collected and stored securely on a secure server at University of Southampton in compliance with the requirements of the General Data Protection Regulations and the Data Protection Act 2018. Data collected on participants' use of the online intervention will be collected automatically by the intervention and stored on secure, firewall protected servers, hosted by the University of Southampton. Only trained research personnel with specific roles within the project will have access to this server. Upon download, usage data will be stored in an encrypted, password protected file, stored on password protected computers. Personal data will be pseudo-anonymised by assigning a participant identifier code (PIC) which will be used to identify the participant during the study. An electronic file linking the PIC to the identifiable patient data will be kept separately in a separate secure place on the University of Southampton server. Only trained

research personnel with specific roles assigned will be granted access to the electronic participant data. At the end of the project, all personal data will be permanently deleted.

### 11.2 Research data

The results of the study will be written up in reports and publications. Anonymised quotations provided by participants during the interviews may be used to illustrate the findings, but participants will not be identifiable.

The anonymised research data (trial master file, transcripts) will be stored for 10 years after the end of the study in accordance with the procedures agreed by the sponsor. During analysis and write-up (approx. 2 years) it will be stored on a secure server or in a locked filing cabinet at University of Southampton, after which it will be stored off site at an approved storage facility that has been agreed by the sponsor. The data custodian is Professor Paul Little, chief investigator.

At the end of the study anonymous questionnaire data will deposited in a secure data archive which will be made available to researchers at University of Southampton for secondary data analysis.

### 11.3 Audio-recordings of participant interviews

Audio-recordings of participant interviews will be collected using a portable digital recording device or using MS Teams. Following each interview, the audio-recordings will be transferred directly to the University of Southampton server (accessible only by members of the study team and University of Southampton IT Services) and then deleted from the digital device. The audio data will be anonymised and identified by a unique participant ID only. Transcribing will be facilitated through a member of the research team or a University-approved third party, using only the participant ID. Transcribers will sign a confidentiality agreement to keep the data confidential; store the data securely; and delete the data when the transcription has been completed and receipt confirmed. The audio-recordings will be permanently deleted on study publication.

## 12 References

1. Ieven M, Coenen S, Loens K, et al. Aetiology of lower respiratory tract infection in adults in primary care: a prospective study in 11 European countries. *Clinical microbiology and infection : the official publication of the European Society of Clinical Microbiology and Infectious Diseases* 2018;24(11):1158-63. doi: 10.1016/j.cmi.2018.02.004 [published Online First: 2018/02/16]

2. Gliklich RE, Metson R. The health impact of chronic sinusitis in patients seeking otolaryngologic care. *Otolaryngol Head Neck Surg* 1995;113(1):104-9. doi: 10.1016/S0194-59989570152-4 [published Online First: 1995/07/01]

3. Witsell DL, Dolor RJ, Bolte JM, et al. Exploring health-related quality of life in patients with diseases of the ear, nose, and throat: a multicenter observational study. *Otolaryngol Head Neck Surg* 2001;125(4):288-98. doi: 10.1067/mhn.2001.118693 [published Online First: 2001/10/11]

4. Andreou N, Hadjisymeou S, Panesar J. Does tonsillectomy improve quality of life in adults? A systematic literature review. *The Journal of laryngology and otology* 2013;127(4):332-8. doi: 10.1017/s0022215113000273 [published Online First: 2013/03/02]

5. Gulliford MC, Dregan A, Moore MV, et al. Continued high rates of antibiotic prescribing to adults with respiratory tract infection: survey of 568 UK general practices. *BMJ Open* 2014;4(10):e006245. doi: 10.1136/bmjopen-2014-006245 [published Online First: 2014/10/29]

6. Gulliford MC, Moore MV, Little P, et al. Safety of reduced antibiotic prescribing for self limiting respiratory tract infections in primary care: cohort study using electronic health records. *Bmj* 2016;354:i3410. doi: 10.1136/bmj.i3410 [published Online First: 2016/07/06]

7. Goossens H, Ferech M, Vander Stichele R, et al. Outpatient antibiotic use in Europe and association with resistance: a cross-national database study. *Lancet* 2005;365(9459):579-87. doi: 10.1016/S0140-6736(05)17907-0 [published Online First: 2005/02/15]

8. Moore M, Little P, Rumsby K, et al. Effect of antibiotic prescribing strategies and an information leaflet on longer-term reconsultation for acute lower respiratory tract infection. *Br J Gen Pract* 2009;59(567):728-34. doi: 10.3399/bjgp09X472601 [published Online First: 2009/10/22]

9. Little P, Gould C, Williamson I, et al. Reattendance and complications in a randomised trial of prescribing strategies for sore throat: the medicalising effect of prescribing antibiotics. *BMJ* 1997;315(7104):350-2. doi: 10.1136/bmj.315.7104.350 [published Online First: 1997/08/09]

10. Little P, Stuart B, Hobbs FD, et al. Antibiotic prescription strategies for acute sore throat: a prospective observational cohort study. *Lancet Infect Dis* 2014;14(3):213-9. doi: 10.1016/S1473-3099(13)70294-9 [published Online First: 2014/01/21]

11. Liu R, Han H, Liu F, et al. Positive rate of RT-PCR detection of SARS-CoV-2 infection in 4880 cases from one hospital in Wuhan, China, from Jan to Feb 2020. *Clinica chimica acta; international journal of clinical chemistry* 2020;505:172-75. doi: 10.1016/j.cca.2020.03.009 [published Online First: 2020/03/12]

12. Kim D, Quinn J, Pinsky B, et al. Rates of Co-infection Between SARS-CoV-2 and Other Respiratory Pathogens. *Jama* 2020 doi: 10.1001/jama.2020.6266 [published Online First: 2020/04/16]

13. Casanova LM, Jeon S, Rutala WA, et al. Effects of air temperature and relative humidity on coronavirus survival on surfaces. *Applied and environmental microbiology* 2010;76(9):2712-7. doi: 10.1128/aem.02291-09 [published Online First: 2010/03/17]

14. Johnson C, Eccles R. Acute cooling of the feet and the onset of common cold symptoms. *Family Practice* 2005;22:608-13.

15. Hughes JH, Thomas DC, Hamparian VV, et al. Acid liability of rhinovirus type 14: effect of pH, time, and temperature. *Proceedings of the Society for Experimental Biology and Medicine Society for Experimental Biology and Medicine (New York, NY)* 1973;144(2):555-60. doi: 10.3181/00379727-144-37634 [published Online First: 1973/11/01]

16. Giranda VL, Heinz BA, Oliveira MA, et al. Acid-induced structural changes in human rhinovirus 14: possible role in uncoating. *Proceedings of the National Academy of Sciences of the United States of America* 1992;89(21):10213-7. doi: 10.1073/pnas.89.21.10213 [published Online First: 1992/11/01]

17. Pratelli A. Canine coronavirus inactivation with physical and chemical agents. *Vet J* 2008;177(1):71-79. doi: 10.1016/j.tvjl.2007.03.019 [published Online First: 05/21]

18. Rennie P, Bowtell P, Hull D, et al. Low pH gel intranasal sprays inactivate influenza viruses in vitro and protect ferrets against influenza infection. *Respir Res* 2007;8:38. doi: 10.1186/1465-9921-8-38 [published Online First: 2007/05/19]

19. Gern JE, Mosser AG, Swenson CA, et al. Inhibition of rhinovirus replication in vitro and in vivo by acid-buffered saline. *J Infect Dis* 2007;195(8):1137-43. doi: 10.1086/512858 [published Online First: 2007/03/16]

20. Little P, Read RC, Amlot R, et al. Reducing risks from coronavirus transmission in the home-the role of viral load. *Bmj* 2020;369:m1728. doi: 10.1136/bmj.m1728 [published Online First: 2020/05/08]

21. Little P, Stuart B, Hobbs FDRea. An internet-delivered handwashing intervention to modify influenza-like illness and respiratory infection transmission (PRIMIT): a primary care randomised trial. *Lancet* 2015;DOI: http://dx.doi.org/10.1016/S0140-6736(15)60127-1

22. Hull D, Rennie P, Noronha A, et al. Effects of creating a non-specific, virus-hostile environment in the nasopharynx on symptoms and duration of common cold. *Acta otorhinolaryngologica Italica* 2007;27:73-77.

23. Eccles R, Winther B, Johnston SL, et al. Efficacy and safety of iota-carrageenan nasal spray versus placebo in early treatment of the common cold in adults: the ICICC trial. *Respir Res* 2015;16:121. doi: 10.1186/s12931-015-0281-8 [published Online First: 2015/10/07]

24. Ludwig M, Enzenhofer E, Schneider S, et al. Efficacy of a carrageenan nasal spray in patients with common cold: a randomized controlled trial. *Respir Res* 2013;14:124. doi: 10.1186/1465-9921-14-124 [published Online First: 2013/11/14]

25. Fazekas T, Eickhoff P, Pruckner N, et al. Lessons learned from a double-blind randomised placebo-controlled study with a iota-carrageenan nasal spray as medical device in children with acute symptoms of common cold. *BMC complementary and alternative medicine* 2012;12:147. doi: 10.1186/1472-6882-12-147 [published Online First: 2012/09/07]

26. Grande AJ, Keogh J, Hoffmann TC, et al. Exercise versus no exercise for the occurrence, severity and duration of acute respiratory infections. *Cochrane Database Syst Rev* 2015(6):Cd010596. doi: 10.1002/14651858.CD010596.pub2 [published Online First: 2015/06/17]

27. Barrett B, Hayney MS, Muller D, et al. Meditation or exercise for preventing acute respiratory infection: a randomized controlled trial. *Ann Fam Med* 2012;10(4):337-46. doi: 10.1370/afm.1376 [published Online First: 2012/07/11]

28. Barrett B, Hayney MS, Muller D, et al. Meditation or exercise for preventing acute respiratory infection (MEPARI-2): A randomized controlled trial. *PLoS One* 2018;13(6):e0197778. doi: 10.1371/journal.pone.0197778 [published Online First: 2018/06/23]

29. Nieman DC, Henson DA, Austin MD, et al. Upper respiratory tract infection is reduced in physically fit and active adults. *British journal of sports medicine* 2011;45(12):987-92. doi: 10.1136/bjsm.2010.077875 [published Online First: 2010/11/03]

30. Cohen S, Tyrrell DA, Smith AP. Psychological stress and susceptibility to the common cold. *N Engl J Med* 1991;325(9):606-12. doi: 10.1056/NEJM199108293250903 [published Online First: 1991/08/29]

31. Doyle WJ, Gentile DA, Cohen S. Emotional style, nasal cytokines, and illness expression after experimental rhinovirus exposure. *Brain, behavior, and immunity* 2006;20(2):175-81. doi: 10.1016/j.bbi.2005.05.005 [published Online First: 2005/07/19]

32. Cohen S, Doyle WJ, Skoner DP, et al. Social ties and susceptibility to the common cold. *Jama* 1997;277(24):1940-4. [published Online First: 1997/06/25]

33. Trueba AF, Ritz T. Stress, asthma, and respiratory infections: pathways involving airway immunology and microbial endocrinology. *Brain, behavior, and immunity* 2013;29:11-27. doi: 10.1016/j.bbi.2012.09.012 [published Online First: 2012/10/09]

34. Stover CM. Mechanisms of Stress-Mediated Modulation of Upper and Lower Respiratory Tract Infections. *Advances in experimental medicine and biology* 2016;874:215-23. doi: 10.1007/978-3-319-20215-0_10 [published Online First: 2015/11/22]

35. Carmody J, Baer RA. Relationships between mindfulness practice and levels of mindfulness, medical and psychological symptoms and well-being in a mindfulness-based stress reduction program. *J Behav Med* 2008;31(1):23-33. doi: 10.1007/s10865-007-9130-7 [published Online First: 2007/09/28]

36. Little P, Stuart B, Hobbs FR, et al. An internet-based intervention with brief nurse support to manage obesity in primary care (POWeR+): a pragmatic, parallel-group, randomised controlled trial. *The lancet Diabetes & endocrinology* 2016;4(10):821-8. doi: 10.1016/s2213-8587(16)30099-7 [published Online First: 2016/07/31]

37. Bradbury K, Steele M, Corbett T, et al. Developing a digital intervention for cancer survivors: an evidence-, theory- and person-based approach. *npj Digital Medicine* 2019;2(1):85. doi: 10.1038/s41746-019-0163-4

38. Anthierens S, Tonkin-Crine S, Cals JW, et al. Clinicians' views and experiences of interventions to enhance the quality of antibiotic prescribing for acute respiratory tract infections. *J Gen Intern Med* 2015;30(4):408-16. doi: 10.1007/s11606-014-3076-6 [published Online First: 2014/11/07]

39. Tonkin-Crine S, Yardley L, Little P. Antibiotic prescribing for acute respiratory tract infections in primary care: a systematic review and meta-ethnography. *J Antimicrob Chemother* 2011;66(10):2215-23. doi: 10.1093/jac/dkr279 [published Online First: 2011/07/19]

40. Yardley L, Williams S, Bradbury K, et al. Integrating user perspectives into the development of a web-based weight management intervention. *Clin Obes* 2012;2(5-6):132-41. doi: 10.1111/cob.12001 [published Online First: 2012/10/01]

41. Geraghty AW, Munoz RF, Yardley L, et al. Developing an Unguided Internet-Delivered Intervention for Emotional Distress in Primary Care Patients: Applying Common Factor and Person-Based Approaches. *JMIR Ment Health* 2016;3(4):e53. doi: 10.2196/mental.5845 [published Online First: 2016/12/22]

42. Cook R, Farewell V. Multiplicity Considerations in the Design and Analysis of Clinical Trials. *J Royal Stats Soc* 1996;Series A 159:93-110.

43. Yardley L, Morrison L, Bradbury K, et al. The person-based approach to intervention development: Application to digital health-related behavior change interventions. *Journal of Medical Internet Research* 2015;17(1)

44. Little P, Williamson I, Warner G, et al. Open randomised trial of prescribing strategies in managing sore throat. *BMJ* 1997;314(7082):722-7. doi: 10.1136/bmj.314.7082.722 [published Online First: 1997/03/08]

45. Little P, Stuart B, Mullee M, et al. Effectiveness of steam inhalation and nasal irrigation for chronic or recurrent sinus symptoms in primary care: a pragmatic randomized controlled trial. *CMAJ* 2016;188(13):940-49. doi: 10.1503/cmaj.160362 [published Online First: 2016/07/20]

46. Craig CL, Marshall AL, Sjostrom M, et al. International physical activity questionnaire: 12-country reliability and validity. *Med Sci Sports Exerc* 2003;35(8):1381-95. doi: 10.1249/01.MSS.0000078924.61453.FB [published Online First: 2003/08/06]

47. Marshall AL, Miller YD, Burton NW, et al. Measuring total and domain-specific sitting: a study of reliability and validity. *Med Sci Sports Exerc* 2010;42(6):1094-102. doi: 10.1249/MSS.0b013e3181c5ec18 [published Online First: 2009/12/10]

48. Cohen S, Kamarck T, Mermelstein R. A global measure of perceived stress. *J Health Soc Behav* 1983;24(4):385-96. [published Online First: 1983/12/01]

49. Kroenke K, Spitzer RL, Williams JB. The PHQ-9: validity of a brief depression severity measure. *J Gen Intern Med* 2001;16(9):606-13. [published Online First: 2001/09/15]

50. Ruiz MA, Zamorano E, Garcia-Campayo J, et al. Validity of the GAD-7 scale as an outcome measure of disability in patients with generalized anxiety disorders in primary care. *Journal of affective disorders* 2011;128(3):277-86. doi: 10.1016/j.jad.2010.07.010 [published Online First: 2010/08/10]

51. Herdman M, Gudex C, Lloyd A, et al. Development and preliminary testing of the new five-level version of EQ-5D (EQ-5D-5L). *Qual Life Res* 2011;20(10):1727-36. doi: 10.1007/s11136-011-9903-x [published Online First: 2011/04/12]

52. Little PS, Williamson I, Warner G, et al. An open randomised trial of prescribing strategies for sore throat. *B M J* 1997;314:722-27.

53. Braun V, Clarke V. Using thematic analysis in psychology. *Qualitative Research in Psychology* 2006;3(2):77-101. doi: 10.1191/1478088706qp063oa

54. Yardley L, Bishop F. Mixing qualitative and quantitative methods: a pragmatic approach. Willig, Carla and Stainton-Rogers, Wendy (eds) The SAGE Handbook of Qualitative Research in Psychology SAGE Publications, pp 352-3702007.

55. Kirby S, Donovan-Hall M, Yardley L. Measuring barriers to adherence: validation of the problematic experiences of therapy scale. *Disability and Rehabilitation* 2014;36(22):1924-29. doi: 10.3109/09638288.2013.876106
