# Supplementary material for: Determining the clinical and cost-effectiveness of nasal sprays and a physical activity and stress management intervention to reduce respiratory tract infections in primary care: A protocol for the ‘Immune Defence’ randomised controlled trial
Source: PLoS One. 2023 Jul 14;18(7):e0285693. doi: 10.1371/journal.pone.0285693 (PMC10348543; doi:10.1371/journal.pone.0285693)
Supplement: Peer review history

# Editor report: Decision Letter 0

**DOI:** 10.1371/journal.pone.0285693.r001

**Authors:** Johannes Stortz

28 Apr 2023

Determining the clinical and cost-effectiveness of nasal sprays and a physical activity and stress management intervention to reduce respiratory tract infections in primary care:  A protocol for the ‘Immune Defence’ randomised controlled trial.

PONE-D-23-09342

Dear Dr. Vennik,

We’re pleased to inform you that your manuscript has been judged scientifically suitable for publication and will be formally accepted for publication once it meets all outstanding technical requirements.

Within one week, you’ll receive an e-mail detailing the required amendments. When these have been addressed, you’ll receive a formal acceptance letter and your manuscript will be scheduled for publication.

An invoice for payment will follow shortly after the formal acceptance. To ensure an efficient process, please log into Editorial Manager at http://www.editorialmanager.com/pone/, click the 'Update My Information' link at the top of the page, and double check that your user information is up-to-date. If you have any billing related questions, please contact our Author Billing department directly at authorbilling@plos.org.

If your institution or institutions have a press office, please notify them about your upcoming paper to help maximize its impact. If they’ll be preparing press materials, please inform our press team as soon as possible -- no later than 48 hours after receiving the formal acceptance. Your manuscript will remain under strict press embargo until 2 pm Eastern Time on the date of publication. For more information, please contact onepress@plos.org.

Kind regards,

Johannes Stortz

Staff Editor

PLOS ONE

Journal Requirements:

1. Please include a separate caption for each figure in your manuscript.

Additional Editor Comments (optional):

Reviewers' comments:

# Editor report: Acceptance letter

**DOI:** 10.1371/journal.pone.0285693.r002

**Authors:** Johannes Stortz

4 Jul 2023

PONE-D-23-09342 

Determining the clinical and cost-effectiveness of nasal sprays and a physical activity and stress management intervention to reduce respiratory tract infections in primary care:  A protocol for the ‘Immune Defence’ randomised controlled trial. 

Dear Dr. Vennik:

I'm pleased to inform you that your manuscript has been deemed suitable for publication in PLOS ONE. Congratulations! Your manuscript is now with our production department. 

If your institution or institutions have a press office, please let them know about your upcoming paper now to help maximize its impact. If they'll be preparing press materials, please inform our press team within the next 48 hours. Your manuscript will remain under strict press embargo until 2 pm Eastern Time on the date of publication. For more information please contact onepress@plos.org.

If we can help with anything else, please email us at plosone@plos.org. 

Thank you for submitting your work to PLOS ONE and supporting open access. 

Kind regards, 

PLOS ONE Editorial Office Staff

on behalf of

Johannes Stortz 

%CORR_ED_EDITOR_ROLE%

PLOS ONE
